# Should I Stop or Should I Go? The Role of Associations and Expectancies

**DOI:** 10.1037/xhp0000116

**Published:** 2015-08-31

**Authors:** Maisy Best, Natalia S. Lawrence, Gordon D. Logan, Ian P. L. McLaren, Frederick Verbruggen

**Affiliations:** 1 School of Psychology, University of Exeter; 2 Department of Psychology, Vanderbilt University; 3 School of Psychology, University of Exeter

**Keywords:** response inhibition, attention, automaticity, associative learning, expectancy

## Abstract

Following exposure to consistent stimulus–stop mappings, response inhibition can become automatized with practice. What is learned is less clear, even though this has important theoretical and practical implications. A recent analysis indicates that stimuli can become associated with a stop signal or with a stop goal. Furthermore, expectancy may play an important role. Previous studies that have used stop or no-go signals to manipulate stimulus–stop learning cannot distinguish between stimulus-signal and stimulus-goal associations, and expectancy has not been measured properly. In the present study, participants performed a task that combined features of the go/no-go task and the stop-signal task in which the stop-signal rule changed at the beginning of each block. The go and stop signals were superimposed over 40 task-irrelevant images. Our results show that participants can learn direct associations between images and the stop goal without mediation via the stop signal. Exposure to the image-stop associations influenced task performance during training, and expectancies measured following task completion or measured within the task. But, despite this, we found an effect of stimulus–stop learning on test performance only when the task increased the task-relevance of the images. This could indicate that the influence of stimulus–stop learning on go performance is strongly influenced by attention to both task-relevant and task-irrelevant stimulus features. More generally, our findings suggest a strong interplay between automatic and controlled processes.

Response inhibition is often considered to be a deliberate act of top-down cognitive control. It allows people to quickly stop and replace actions that are no longer relevant or that are inappropriate in the current task environment. Longitudinal studies have shown that response inhibition and self-control in childhood and adolescence correlate with a variety of life outcomes in adulthood, including personal finances and engagement in healthy behaviors (Diamond, 2013; Moffitt et al., 2011; Nigg et al., 2006). Furthermore, clinical research suggests that impairments in response inhibition may contribute to the development of a range of psychopathological and impulse-control disorders, such as attention-deficit/hyperactivity disorder, obsessive–compulsive disorder, substance abuse, pathological gambling, and eating disorders (Bechara, Noel, & Crone, 2006; Crews & Boettiger, 2009; de Wit, 2009; Fernie et al., 2013; Garavan & Stout, 2005; Nigg, 2001; Noël, Brevers, & Bechara, 2013). Response inhibition efficiency also correlates with the treatment outcome in people with such disorders (e.g., Nederkoorn, Jansen, Mulkens, & Jansen, 2007). Thus, the ability to stop actions seems very important for adaptive and goal-directed behavior. However, in recent years, research has demonstrated that response inhibition may not always be the executive, deliberate, act of control that it is typically assumed to be. In the present study, we will further explore the interplay between “bottom-up” and “top-down” control processes when stopping a response.

Popular paradigms used to study response inhibition in healthy and clinical populations are the go/no-go task (Donders, 1868/1969) and the stop-signal task (Logan & Cowan, 1984; Verbruggen & Logan, 2008c). Research using these tasks has demonstrated both short-term and long-term aftereffects of stopping (e.g., Bissett & Logan, 2011; Enticott, Bradshaw, Bellgrove, Upton, & Ogloff, 2009; Giesen & Rothermund, 2014; Rieger & Gauggel, 1999; Verbruggen & Logan, 2008b; Verbruggen, Logan, Liefooghe, & Vandierendonck, 2008). For example, responding to a stimulus is typically slowed after a stop-signal trial. This slowing is more pronounced when the primary-task stimulus of the previous stop-signal trial is repeated, which has led to the suggestion that people can learn associations between specific stimuli and stopping (Rieger & Gauggel, 1999; Verbruggen & Logan, 2008b; Verbruggen et al., 2008). The idea that a specific stimulus can become associated with stopping is consistent with studies that have highlighted the role of stimulus–response (s–r) bindings in other cognitive control paradigms, such as the negative priming paradigm (cf. the “do-not-respond” tag account; Neill, Valdes, Terry, & Gorfein, 1992; see also Neill & Valdes, 1992), the task-switching paradigm (e.g., Koch & Allport, 2006; Waszak, Hommel, & Allport, 2003, 2004, 2005), and interference control tasks (e.g., Anderson & Folk, 2014; Hommel, Proctor, & Vu, 2004). The formation of stimulus–stop (s–s) bindings may be the first step toward automaticity (Logan, 1990). Memory-retrieval accounts of automatization assume that every time people respond to a stimulus, processing episodes are stored as “instances” (Logan, 1988) or “event files” (Hommel, 1998, 2004) in memory. These instances or event files may contain information about the stimulus (e.g., the word), the interpretation given to the stimulus (e.g., “natural”), the task goal (e.g., “go”) and the response (e.g., “left key press”).^1^ These episodes are retrieved when a stimulus is repeated and will influence responding. For example, the instance theory (Logan, 1988) postulates that action selection can be construed as a race between an algorithmic response-selection process and a memory-retrieval process; the process that finishes first determines which action is selected. When the memory-retrieval process wins the race, the decision is said to be automatic, whereas decisions based on algorithmic processing are deliberate or intentional (Logan, 1988). Therefore, when the s–r or s–s mapping is the same throughout practice, multiple instances are formed and automatic processing can develop (Logan, 1988; Shiffrin & Schneider, 1977).

Some of us examined the idea that inhibitory control in go/no-go and stop-signal tasks can be triggered automatically via the retrieval of s–s associations from memory (Verbruggen & Logan, 2008a). For example, in a series of go/no-go experiments, a stimulus category determined if a participant should respond or not (e.g., living word referents = go; nonliving word referents = no-go). After a training phase, the go/no-go mapping was reversed in a test phase. We found that responding to the old stop stimuli was slowed compared with new stimuli that were not previously presented during training (consequently, these new stimuli were not associated with going or stopping) or with old stimuli that were associated with going. This response slowing was also found in modified versions of the stop-signal task in which the contingencies between specific go stimuli and stopping were manipulated, such that certain items were consistently presented on stop-signal trials, whereas other items were presented on both go and stop-signal trials. Consistent with the go/no-go results, we found that responding was slowed for old stop items compared with inconsistent items that were not particularly associated with going or stopping (Lenartowicz, Verbruggen, Logan, & Poldrack, 2011; Verbruggen & Logan, 2008a). Furthermore, the Lenartowicz et al. (2011) study demonstrated that old stop items activated the neural stopping network. Thus, response inhibition may become automatized after sufficient practice with consistent s–s mappings (Jasinska, 2013; Lenartowicz et al., 2011; Spierer, Chavan, & Manuel, 2013; Verbruggen, Best, Bowditch, Stevens, & McLaren, 2014; Verbruggen & Logan, 2008a). These findings may have important implications for our current theories of response inhibition and executive control. Furthermore, they could also have practical applications. Recent studies suggest that the acquisition of s–s associations could be an effective way to reduce engagement in impulsive behaviors, such as excessive food (e.g., Houben & Jansen, 2011) and alcohol (e.g., Jones & Field, 2013) consumption. These studies used paradigms in which no-go or stop signals were superimposed over, or presented around, images of unhealthy foods or alcohol (e.g., Bowley, Faricy, Hegarty, Johnstone, & Smith, 2013; Houben & Jansen, 2011; Houben, Nederkoorn, Wiers, & Jansen, 2011; Houben, 2011; Jones & Field, 2013; Lawrence, Verbruggen, Morrison, Adams, & Chambers, 2015; Veling, Aarts, & Papies, 2011; Veling, Aarts, & Stroebe, 2013; Veling, van Koningsbruggen, Aarts, & Stroebe, 2014). Pairing these images with stopping reduced subsequent consumption of unhealthy foods and alcohol. Therefore, this research suggests that automatic inhibition could be useful in the treatment of a variety of impulse-control disorders (for a recent meta-analysis, see Jones et al., 2015).

Current research in the stop-learning literature appears to provide strong support for the ‘automatic inhibition’ account that postulates that stimuli can become associated with the act of stopping. However, a recent review indicates that it is still unclear exactly what is learned in these tasks and how this influences performance (Verbruggen, Best et al., 2014). The present study was designed to address two of the main outstanding issues that we highlighted in our review (similar issues were also recently raised in the context of s-r bindings; Henson, Eckstein, Waszak, Frings, & Horner, 2014): (1) are associations between stimuli and stopping direct, and (2) to what extent does expectancy play a role?

## Are Associations Between Stimuli and Stopping Direct?

The automatic inhibition account assumes that people learn direct associations between a stimulus and the act of stopping in go/no-go tasks and modified versions of the stop-signal task. However, the results of a recent experiment are inconsistent with this account (Verbruggen, Best et al., 2014). In that experiment, participants made speeded semantic categorizations (living/nonliving) of a series of words. On some trials (stop-signal trials) an additional visual signal was presented below the word, instructing participants to withhold their planned response. Certain words were consistently presented on stop-signal trials, whereas other words were presented on go and stop-signal trials with equal probability. We found that the probability of responding on stop-signal trials was lower for the consistent words than for the inconsistent words in the training phase, indicating that learning had occurred. However, we found no difference in go reaction time (RT) between the old stop words and the inconsistent words when the s–s mapping was subsequently reversed in the test phase. In other words, learning influenced stop performance on signal trials in the training phase, but it did not influence go performance on no-signal trials in the test phase. We proposed that this pattern of results indicates that participants learned stimulus-signal associations rather than s–s associations. Such associations between the stop words and the stop signal (i.e., the line turning bold) will prime the representation of the stop *signal* rather than the stop *goal*. Signal detection plays a critical role in successful stopping (e.g., Verbruggen, Stevens, & Chambers, 2014), and computational work suggests that a considerable proportion of the stopping latency is occupied by perceptual or afferent processes (Boucher, Palmeri, Logan, & Schall, 2007; Logan, Van Zandt, Verbruggen, & Wagenmakers, 2014; Logan, Yamaguchi, Schall, & Palmeri, 2015; Salinas & Stanford, 2013). Thus, by priming the representation of the stop signal, learning could lead to improvements in stopping performance on stop-signal trials without influencing responding on go trials in the test phase.

The idea that participants could learn stimulus-signal associations is also consistent with a range of research on learning and conditioning in humans and other animals that indicates that stimulus detection can itself become conditioned (McLaren, Wills, & Graham, 2010) and, of course, that links between perceptual stimuli can be established. As an illustrative (and rather basic) example, in a classic autoshaping paradigm with pigeons, the presentation of a conditioned stimulus (e.g., a keylight) and an unconditioned stimulus (e.g., the delivery of food) usually co-occur. With practice, the presentation of the conditioned stimulus alone can come to elicit the conditioned response (e.g., pecking at this key). The conditioned stimulus can activate this response via two routes; either indirectly via the conditional stimulus (CS)-US link, or more directly, via a CS-R link (Hall, 2002). Thus, it seems plausible that learning can also influence perception of the no-go or stop signal in response inhibition paradigms.

**Figure 1 fig1:**
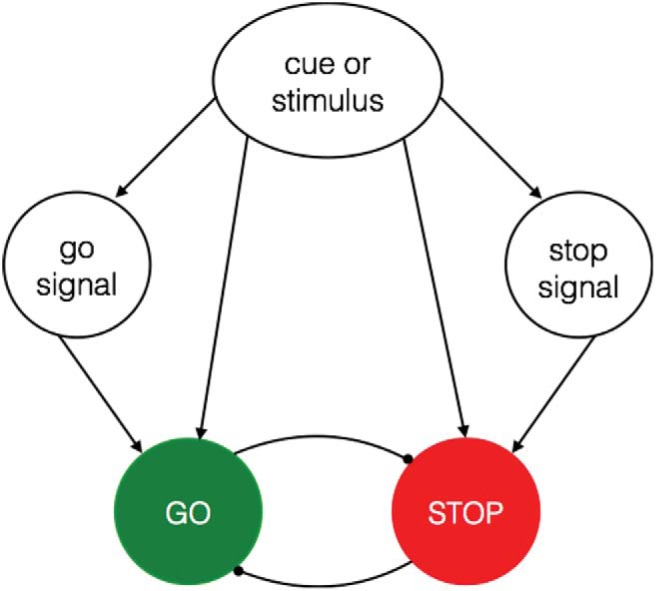
Overview of the architecture of the associative stop system (for a more detailed overview, see Verbruggen, Best, et al., 2014). There are two associative routes to activating the stop-goal; a direct association between the stimulus or cue and the go/stop goal, or indirect association between the stimulus or cue and the go/stop goal that is mediated via a representation of the go/stop signal. Excitatory and inhibitory connections are represented on the diagram with arrows. See the online article for the color version of this figure.

The potential for stimulus-signal associations has important implications for the interpretation of previously reported behavioral effects in the stop-learning literature. Previous studies that have used no-go or stop signals to manipulate s–s learning cannot distinguish between stimulus-goal and stimulus-signal learning. It is therefore possible that previously observed RT effects and neural activations (Lenartowicz et al., 2011; Manuel, Bernasconi, & Spierer, 2013; Manuel, Grivel, Bernasconi, Murray, & Spierer, 2010) could be mediated by a link between the stimulus, the stop signal, and stopping (see Figure 1). Similarly, in go/no-go experiments in which the go/no-go rules are explicit (e.g., living = go, nonliving = no-go), the s–s association could be mediated via the go/no-go category (e.g., ‘desk = nonliving -> nonliving = no-go,’ instead of ‘desk = no-go’). In addition to being of theoretical interest, the idea of s–s associations also has implications for applied stop-training research (see above). Therefore, in the present study, we investigated whether there is any evidence for the original idea (i.e., as suggested by Verbruggen & Logan, 2008a) that direct associations can be acquired between a stimulus and the stop goal, without mediation via a representation of the stop signal (or no-go category). To discourage the formation of stimulus-signal associations, we changed the stop signal and the task rules at the beginning of each block. The demonstration of response slowing for consistent stop items in the present experiment would provide the strongest evidence to date for the direct s–s hypothesis.[Fig-anchor fig1]

## What Is the Role of Expectancy in s–s Learning?

In the associative-learning literature, there is an ongoing debate surrounding the involvement of explicit and implicit processes in the acquisition of stimulus-action associations (Mitchell, De Houwer, & Lovibond, 2009). To make a broad distinction, ‘explicit’ processes are assumed to be controlled, intentional, effortful, and rule-based; by contrast, ‘implicit’ processes are assumed to be automatic, effortless, and associative (e.g., McLaren, Green, & Mackintosh, 1994; for a recent discussion of the distinction between associative and propositional processes, see McLaren et al., 2014). Expectancy ratings have been used to dissociate between the two processes (e.g., McLaren et al., 2014; Newell & Shanks, 2014). In the context of stop-learning, this dissociation between rule-based processes and associative (s-s or s-r) processes has important theoretical implications. After all, expectancy of a stop signal for old stop items could indicate that the response slowing observed for old stop items is due to proactive inhibitory control, rather than ‘automatic inhibition.’ When a cue indicates that a stop signal is likely to occur on the following trial(s), participants proactively increase response thresholds or suppress motor activation (e.g., Jahfari et al., 2012; Ramautar, Kok, & Ridderinkhof, 2004; Verbruggen & Logan, 2009; Zandbelt, Bloemendaal, Neggers, Kahn, & Vink, 2013). Stimuli associated with stopping could act as such cues (e.g., ‘if stimulus X then *p*(stop) is high’), and participants would adjust their response strategies accordingly. In other words, slowing for old stop items could be due to proactive control (which may be conceived as another ‘algorithmic’ process; cf. Logan, 1988), rather than to the direct activation of the stop response via memory retrieval. The role of expectancy-driven processes is also relevant for the applied stop-training research. Indeed, the extent to which training effects like these reflect implicit or associative effects has been called into question. For example, Boot, Simons, Stothart and Stutts (2013) argued that many ‘control’ training effects could be due to changes in expectations and demand characteristics. The involvement of expectancies would have implications for the longevity of these inhibitory control training effects and the variability of training efficacy across individuals (cf. Boot et al., 2013).

In the present study we investigated the role of expectancy in s–s learning via the inclusion of an additional dependent variable that was sensitive enough (Newell & Shanks, 2014) to detect s–s learning following task completion (Experiments 1–3) or within the task (Experiment 4).

## Experiment 1

In Experiment 1, we combined features of a go/no-go task and a stop-signal task. In standard go/no-go tasks only one stimulus is presented on each trial, determining whether participants have to respond or not. In standard stop-signal tasks participants respond to each stimulus, unless an extra stop signal is presented after a variable delay. In Experiment 1, we used a go/stop task based on those used in studies examining the effects of no-go training effects on food and alcohol consumption (see above). Similar to picture–word Stroop tasks (see, e.g., MacLeod, 1991), go and stop signals were superimposed over 40 neutral images. The delay between the presentation of the images and the signals was 0 ms. A subset of the images was consistently associated with stop signals, another subset was consistently associated with go signals, and the remaining images were control images (not particularly associated with go or stop). After 12 training blocks, the image mappings were reversed, and participants had to respond to the stop-associated images. Participants were not informed about the image mappings, but they were told at the beginning of each block what the go and stop signals were. To discourage the formation of stimulus-signal or stimulus-category associations, we varied the representation of the go and stop signals at the beginning of each block. We predicted that this change manipulation would encourage the formation of image-stop associations (cf. Verbruggen & Logan, 2008a) instead of image-signal associations. We indexed learning during the task via two measures. The first index was the probability of responding on the stop trials, *p*(respond|stop), which was predicted to be lower for stop-associated images than for the control images. The second index was RT on go trials, which was predicted to be longer for the stop-associated images than for the control images. To examine the role of expectancy in stop learning, participants were asked to rate the extent to which they expected to withhold their response for each of the images presented in the task at the end of the experiment.

### Method

#### Subjects

Thirty-one students from the University of Exeter participated for monetary compensation (£5 approximately $7.80) or partial course credit (*M* = 19.43 years, *SD* = 1.70 years, 17 females, 27 right-handed). Two participants were excluded because they incorrectly executed a response on ≥30% of the stop-signal trials (there was no delay between the presentation of the image and the stop signal; consequently, *p*(respond|stop) was expected to be low). The target sample and exclusion criteria were determined before data collection. The data with these participants included are available in the online supplemental material.

#### Apparatus and stimuli

The experiment was run on an Apple iMac using Psychtoolbox (Brainard, 1997). The stimuli were presented on a 20-in monitor (with a 1680 × 1050 resolution). The experimental paradigm consisted of a go/stop task in which the go/stop rule changed at the beginning of each block. The go and stop signals (a full list of the signals used appears in the Appendix A) were superimposed over 40 task-irrelevant neutral images (size: 250 × 250 pixels), which were presented in the center of the screen on a white background. Each image was presented twice per block. In each block, we used two go signals (e.g., the vowels *a* or *e*) and two stop signals (e.g., the consonants *t* or *n*). Participants responded on go trials by pressing the spacebar on a keyboard with their right index finger; they were instructed to withhold their response on stop trials. The signals and the go/stop mapping were shown on the screen at the beginning of each block for a minimum of 5 s, and participants had to press a key to start the first trial. The order of the task rules was randomized across the blocks and the response-rule category was counterbalanced across participants (e.g., go = vowels, stop = consonants vs. go = consonants, stop = vowels).

#### Procedure

Unbeknownst to the participants, there were two phases in the experimental paradigm that determined the image-go/stop mappings; the first 12 blocks of 80 trials comprised the training phase, and the final two blocks of 80 trials comprised the test phase. Participants were verbally instructed to read the task rule screen carefully before starting each block. There was a 15 s break between each block.

There were three image types (see Table 1). First, *stop-associated images* were paired with a stop signal on 75% of presentations in the training phase; in the test phase, they were always paired with a go signal. Second, *go-associated images* were always paired (100%) with a go signal in the training phase, but they could occur on stop trials in the test phase (eight old go-associated images were paired with a stop signal on 75% of presentations; eight old go-associated images were never paired with a stop signal). Third, *control images* were paired with a stop signal on 25% of presentations in the training and test phases. The control images were mostly paired with a go signal during training to ensure that the overall probability of a stop trial (*p*[stop-signal] = 0.25) was the same in the training and the test phases (stopping performance is sensitive to minor variations in signal probability, e.g., see Bissett & Logan, 2011). [Table-anchor tbl1]

**Figure 2 fig2:**
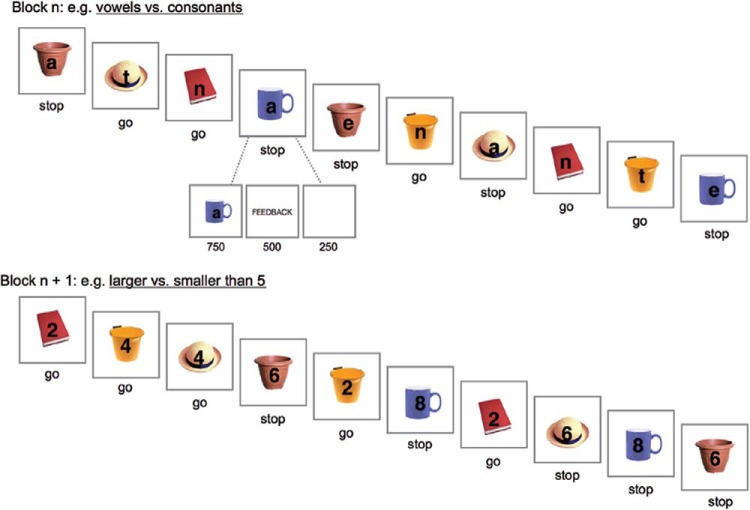
Example go/stop trial sequence. The task rule changed at the beginning of each block (e.g., Block *n*: vowel = stop, consonant = go; Block *n* + 1: > 5 = stop, < 5 = go). In Experiments 1 through 3, the go/stop signals were superimposed on top of the image (as shown). In Experiment 4, the signals were presented in one of the four corners of the image (top-left, bottom-left, top-right, bottom-right). See the online article for the color version of this figure.

All trials began with the concurrent presentation of the image and a go/stop signal (see Figure 2), instructing participants to execute (go) or withhold (stop) the spacebar response. After 750 ms (regardless of RT), the images and go/stop signal were replaced by a feedback message (“correct,” “incorrect,” or “too slow” in case they did not respond before the end of the trial) which remained on the screen for 500 ms. The feedback message was presented to encourage fast and accurate responding. Following the feedback message, there was a blank screen for 250 ms, after which the next trial started.[Fig-anchor fig2]

Following completion of the experimental task, each image was again presented on the screen. The order of the images was randomized anew for each participant. Participants were asked to rate “How much do you expect to withhold your response when this image is presented?” on a scale, ranging from 1 (*I definitely do not think this image indicates that I have to withhold my response*) to 9 (*I definitely think this image indicates that I have to withhold my response*). As a manipulation check, we also asked participants to rate how much they expected to respond (i.e., go) to each of the images (the order of the respond/withhold ratings was counterbalanced across participants). These go ratings were consistent with the stop expectancy ratings so are not reported further.

#### Analyses

All data processing and analyses were completed using R (R Development Core Team, 2013). The training and test phase trials were analyzed separately using analyses of variance (ANOVA) with image type and block as within-subjects factors. Performance was assessed in terms of average RT for correct go responses, the probability of a missed go response [*p*(miss)] and the probability of responding on a stop trial [*p*(respond|stop)]. RTs <1 ms were removed prior to analysis. We did not analyze *p*(miss) further as values were very low (see Table 2). Table 3 provides an overview of the ANOVAs. For pairwise comparisons, Hedge’s g_av_ is the reported effect size measure (Lakens, 2013). All data files and R scripts used for the analyses are deposited on the *Open Research Exeter* data repository (http://hdl.handle.net/10871/17735).[Table-anchor tbl2][Table-anchor tbl3]

### Results

#### Training phase

**Figure 3 fig3:**
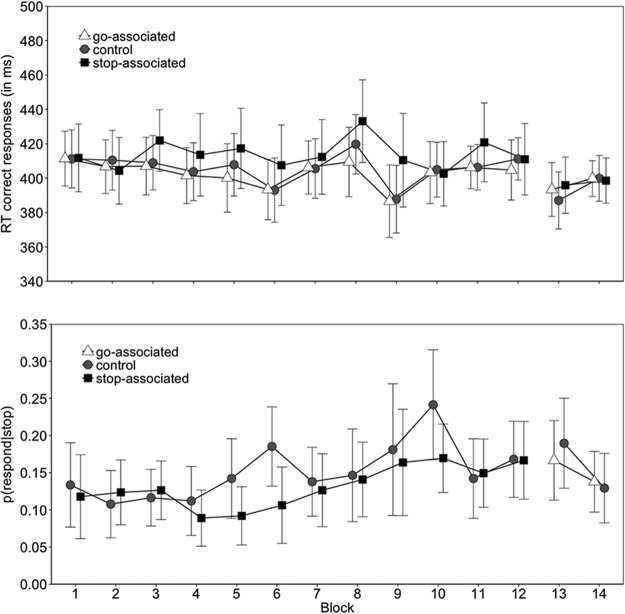
Go reaction times (RTs; upper panel) and *p*(respond|stop) data (lower panel) for the three image types (stop, go, control) as a function of the block (Blocks 1–12 = training phase; Blocks 13–14 = test phase) in Experiment 1. Error bars are 95% confidence intervals.

The main effect of image type on go RTs was reliable (*p* < .001); planned comparisons revealed that responding to the stop-associated images (on the relevant 25% of trials) was slower (414 ms) than to the go-associated images (403 ms), *t*(28) = −4.93, *p* < .001, g_av_ = 0.440, and to the control images (406 ms), *t*(28) = −3.26, *p* = .002, g_av_ = 0.327. There was a marginally reliable difference between the go and the control images, *t*(28) = −1.99, *p* = .055, g_av_ = 0.109 (Figure 3; Table 3). In line with our predictions, the *p*(respond|stop) was lower for the stop-associated images (0.131) than for the control images (0.151), *p* = .019 (see Figure 3). Thus, performance on go and stop trials suggests that participants acquired the image-stop associations. The effect of block and the interaction between block and image type did not reach significance, suggesting that the effect of image type was present in most blocks (see Table 3). This is consistent with our previous work, which indicates that the effect of stop learning emerges after a single trial presentation, and that it then quickly asymptotes (Verbruggen & Logan, 2008a; Verbruggen & Logan, 2008b). The absence of an overall practice effect is most likely due to the introduction of a novel go/stop rule at the beginning of each block; consistent with this idea, a post hoc test confirmed that participants responded faster in the second half of a block than in the first half, *t*(28) = 3.99, *p* < .001, g_av_ = 0.324.[Fig-anchor fig3]

#### Test phase

In the test phase, the stop-associated images were always paired with a go signal, the control images were paired with a stop signal on 25% of the trials (i.e., the control images remained the same in the training and test phases), and the go-associated images were mostly paired with a stop signal (see Table 1). On the basis of the automatic inhibition hypothesis, we predicted that responding on go trials would be slower for the stop-associated images than for the go-associated images and for the control images. Furthermore, *p*(respond|stop) should be higher for the go-associated images than for the control images. However, image type did not influence RT nor *p*(respond|stop) in the test phase (*p*s ≥ 0.557; Table 4). It is possible that the absence of the test phase effect is due to differences in the overall RT (as RTs were faster in the test phase than in the training phase). To investigate this possibility, we plotted RT percentiles for the training and test phases. This revealed that the overall test phase RT cannot account for the absence of the predicted image-stop learning effects (see online supplemental material).[Table-anchor tbl4]

#### Expectancy ratings

Due to technical reasons, one participant in Experiment 1 did not complete the expectancy ratings task. The results of the test phase raise some doubts about whether participants learned long-term image-stop associations. However, the analysis of the expectancy ratings obtained following task completion revealed a main effect of image type, *F*(2, 54) = 10.06, *p* < .001, *gen. η^2^* = 0.075. Consistent with the s–s contingencies during training, participants expected to withhold their response more when the stop-associated images were presented (4.83) than when the go-associated images (3.91) and the control images (4.26) were presented; *t*(27) = −3.46, *p* = .001, g_av_ = 0.653 and *t*(27) = −2.74, *p* = .010, g_av_ = 0.403, respectively. The difference between the control and the go-associated images was also reliable, *t*(27) = −2.89, *p* = .007, g_av_ = 0.271. Thus, participants could distinguish between the images on the basis of their association with the stop and go goals. The stop-minus-control image expectancy difference correlated with the corresponding RT difference in the test phase, *r*(26) = 0.437, *p* = .019: participants who expected to withhold their response more during the presentation of the old stop-associated images slowed more when they had to respond to these images in the test phase. This suggests that expectancies generated on the basis of the acquired image-stop mappings may contribute to the manifestation of an automatic inhibition effect in the test phase. However, there was no reliable correlation between the stop-minus-control expectancy difference and the corresponding RT in the training phase, *r*(26) = 0.010, *p* = .961. There was also no reliable correlation between the RT and expectancy differences for the stop- and the go-associated images in the training phase, *r*(26) = - 0.040, *p* = .841, or the test phase, *r*(26) = 0.272, *p* = .161. (Note that uncorrected *p*s are reported.)

### Discussion

In Experiment 1, we investigated two questions highlighted in our recent review article (Verbruggen, Best, et al., 2014): (1) can participants learn direct associations between stimuli and stopping and (2) what is the role of expectancy in s–s learning? The results provide some answers to both questions. Task performance during the training phase showed that participants could acquire direct s–s associations when the rules (and consequently, signals) constantly changed throughout the task. This indicates that the learning effects were not mediated via signal representations (as each image was only presented twice per block and there were two stop signals and two go signals per block). Furthermore, the expectancy data obtained following task completion showed that participants generated expectancies that were consistent with the s–s contingencies acquired during training.

However, the results of Experiment 1 raised a new question: why did the s–s associations not influence performance in the test phase? We found an associative effect on behavior that appeared early in training but then disappeared again in the later training blocks and in the test phase (Figure 3; for similar results in another action control paradigm, see Gaschler & Nattkemper, 2012), even though the expectancy data measured at the end of the experiment indicated that the associations were not forgotten. We attribute this to an interaction between attention and learning. The role of attention in s–s learning has not yet been considered (and, indeed, is something we did not discuss in our recent review; Verbruggen, Best et al., 2014). In previous studies demonstrating s–s learning (e.g., Verbruggen & Logan, 2008a), the go/stop items were task-relevant as they determined the required response; consequently, optimal task performance in these studies depended on participants attending to the stop items (as opposed to the signals). In the present study, we adapted a paradigm frequently used in applied research (e.g., Houben & Jansen, 2011), whereby go/stop signals were superimposed on a series of images. This was advantageous as it allowed us to vary the representation of the go/stop signals throughout the task while independently manipulating the image-stop contingencies. However, a consequence of this procedure is that optimal task performance does not depend on attending to the stop-associated images. Initially, the task-irrelevant images may have captured attention because they were novel, allowing the effects of learning to emerge. But habituation to the images and reduced salience may have reduced attentional capture, and consequently, weakened or even eliminated the effects of stop-learning on behavior in later blocks.

The hypothesized role of attention in the acquisition of s–s associations is consistent with the associative learning literature. For example, a review by Kruschke (2003) indicates that attention is crucial in explaining associative learning phenomena. Following the principles first enunciated by Mackintosh (1975), he argued that attending to informative cues while ignoring irrelevant cues will accelerate learning. Furthermore, the amount of attention that is paid to the cues will determine the influence of acquired associations on behavior. In a similar vein, instance theory assumes that attention determines what is learned and what is retrieved (Logan & Etherton, 1994; Logan, 1988). But attention can also be influenced by learning. For example, the learned predictability of the outcome relative to other concurrently presented cues may influence the extent to which cues are considered informative or salient, and consequently, the extent to which participants attend to them (see Mackintosh, 1975). Consistent with this suggestion, Livesey and McLaren (2007) demonstrated that stimuli that were better predictors of an outcome became relatively more salient than stimuli that were worse predictors of the outcome over practice (see also Le Pelley & McLaren, 2003).^2^ In other words, previous research indicates that attention and associative learning go hand in hand.

In Experiment 1, the stop-associated images could be considered relatively worse predictors of the stop goal when presented with a stop signal. After all, the stop-associated images were associated with the stop goal (i.e., the outcome in this case) on 75% of the trials, whereas any given stop signal (e.g., the consonants *t* or *n*) was associated with the stop goal on 100% of presentations. Similarly, control images could occur on both go and stop trials. Therefore, attentional accounts of associative learning predict that the images would decrease in salience with exposure; consequently, their contribution to performance would also diminish with increased image exposure (see Le Pelley, Suret, & Beesley, 2009). The suggestion that the relative salience of the images diminished during training is also consistent with conflict monitoring accounts (e.g., Botvinick, Braver, Barch, Carter, & Cohen, 2001). These accounts predict decreased attention to the images due to response conflict triggered by the inconsistency in the predictability of these images. For instance, Egner and Hirsch (2005) have demonstrated that when response conflict is detected, task-relevant information is amplified. Hence, conflict detection accounts predict that participants should increase their attention to the go/stop signals relative to the task-irrelevant images. Thus, in this regard, the main difference between the associative learning and conflict monitoring accounts is the detailed mechanism by which the cognitive system adjusts attentional settings. The conflict account requires conflict to drive this change in attention whereas the associability account does not. All the latter requires is that one stimulus (in this case the stop signal itself) has a greater associative strength to the outcome (stopping) than the other stimulus present (the image).

In sum, the findings of Experiment 1 show that participants can acquire direct associations between specific stimuli and the stop goal. However, despite reliable learning effects in the training phase and in expectancy ratings obtained following task completion, we found no evidence of learning in the test phase when the s–s mappings were reversed. We hypothesize that attention plays a role in determining the influence of s–s learning on behavior. This idea could put important constraints on current theories of the automaticity of control processes. Therefore, we conducted three more experiments to replicate and extend the findings of Experiment 1, and to explore the role of attention in the influence of s–s associations on behavior.

## Experiment 2

In Experiment 1, we hypothesized that habituation and the predictability of the signal-stop contingency relative to the image-stop contingency decreased the amount of attention that was paid to the stop-associated images over practice. To investigate the predictability hypothesis, in Experiment 2, we manipulated the contingency between the images and stopping, to ensure that the stop-associated images were paired with a stop signal and were predictive of the stop goal on 100% of presentations during training (cf. 75% of presentations in Experiment 1). This should prevent conflict driving down attention, but it would not abolish any associability effects as the stop signal would still tend to be the stimulus with the strongest connection to stopping. All that an associability theory requires for the images to lose attention is that they are worse predictors of the outcome relative to the stop signal(s). This will occur when the stop signal(s) always predicts the outcome whereas the images only predict the stop goal on the trials on which they occur. As a result, image associability will be driven down in a block, and will not have time to recover when the stop signal changes at the beginning of each block.

### Method

#### Subjects

Thirty students from the University of Exeter participated for monetary compensation (£5, approximately $7.80) or partial course credit (*M* = 19.97 years, *SD* = 2.81, 23 females, 27 right-handed). No participants were excluded.

#### Apparatus, stimuli, procedure, and analyses

The apparatus, stimuli, and procedure were identical to those of Experiment 1, except for the following changes: the stop-associated images (10 images) were paired with a stop signal on 100% of trials during the training phase and were never paired with a stop signal in the test phase; the go-associated images (30 images) were never paired with a stop signal in the training phase, but some of these images were paired with a stop signal in the test phase (20 old go-associated images were never paired with a stop signal; 10 old go-associated images were paired with a stop signal on 100% of the trials). The analyses were identical to those of Experiment 1, except that the contingencies meant that, for obvious reasons, we could not examine the effect of image type on go RTs or *p*(respond|stop) in the training phase of this experiment (see Table 1).

### Results

#### Training phase

**Figure 4 fig4:**
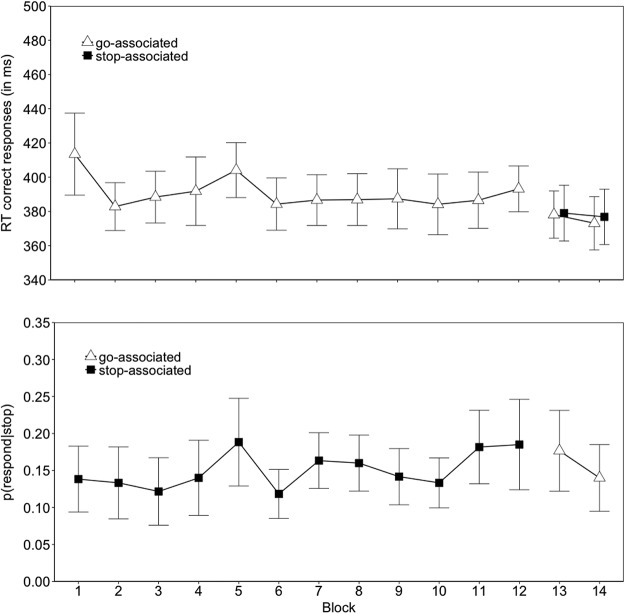
Go reaction times (RTs; upper panel) and *p*(respond|stop) data (lower panel) for the two image types (stop, go) as a function of the block (Blocks 1–12 = training phase; Blocks 13–14 = test phase) in Experiment 2. Error bars are 95% confidence intervals.

In the training phase, the RT for the go-associated images reliably decreased as a function of block (*p* = .038). This suggests that participants acquired the stimulus-go associations during the training. The *p*(respond|stop) for the stop-associated images did not reliably decrease as a function of practice (Figure 4; Table 3), which could be the result of a floor effect.[Fig-anchor fig4]

#### Test phase

Contrary to the predictions of the automatic inhibition hypothesis, go RT was not influenced by image type in the test phase when the image-stop mappings were reversed (see Table 4). As in Experiment 1, the absence of an effect in the test phase cannot be accounted for by the overall speeding of RTs (for RT distributions, see the online supplemental material).

#### Expectancy ratings

Despite the absence of an effect of image-stop learning in the test phase, expectancy ratings obtained following task completion revealed a main effect of image type: participants expected to withhold their response more for the stop-associated images (5.99) than for the go-associated images (3.86), *t*(29) = −5.17, *p* < .001, g_av_ = 1.436. This suggests that participants had learned the image-stop contingencies during training, even though these contingencies did not significantly influence performance in the test phase. The “stop-minus-go” image expectancy difference did not significantly correlate with the RT difference in the test phase, *r*(28) = 0.262, *p* = .162. Note that the stop-minus-go expectancy difference was larger in Experiment 2 than in Experiment 1 (in which stop items could occur on 25% of go trials in the training phase), *t*(49) = −2.47, *p* = .017, Cohen’s *d* = 0.644. In other words, this between-experiment comparison indicates that the image-stop contingency (100% in Experiment 2 relative to 75% in Experiment 1) influenced expectancy ratings but it did not influence performance during the test phase.

### Discussion

In Experiment 2, we investigated whether the relative predictability of the stop-associated images influenced the extent to which the acquired s–s associations influenced task performance when these mappings were reversed. Therefore, the stop-associated images were paired with stopping on 100% of presentations during training (cf. 75% of presentations in Experiment 1).

Consistent with Experiment 1, the decrease in go RT for the go-associated images shows that participants acquired the image-go associations during training (i.e., they associated the go-associated images with responding), and the expectancy ratings obtained following task completion show that participants expected to stop their responses more for the stop-associated images than for the go-associated images. Furthermore, these expectancy ratings were sensitive to the increased predictability of the stop-associated images as the expectancy difference between stop-associated and go-associated images was larger in Experiment 2 than in Experiment 1. However, as in Experiment 1, RTs were comparable for the old stop-associated images and the old go-associated images in the test phase, which indicates that the acquired associations did not influence performance in the test phase when the image-stop mappings reversed. On the face of it, these results do not support the conflict account of attentional modulation (e.g., Botvinick et al., 2001). However, it is possible that participants quickly learned to ignore the images in the test phase when the mapping had reversed. Consistent with this idea, participants were slower to respond to the stop-associated images (382 ms) than to the go-associated images (376 ms) in the first half of Block 13, but this was in the opposite direction in the second half of Block 13 (stop-associated images: 374 ms; go-associated images: 380 ms. This reversal could be due to an increased error signal in the first half of the test phase). This suggests that participants may have quickly relearned the new mappings in the test phase. Note that we did not conduct any inferential statistics on this difference due to low numbers of trials (≤20 trials per cell). An alternative possibility is that participants habituated to the images and stopped paying attention to them because the images were less novel. We tested the habituation hypothesis in Experiment 3.

## Experiment 3

The aim of Experiment 2 was to investigate whether the relative predictiveness of the stop-associated images influenced the extent to which the s–s mappings acquired during training influenced task performance in the test phase. However, even though participants acquired the s–s mappings, these mappings did not modulate performance in the test phase. It is possible that the predictability manipulation did not prevent participants ‘tuning-out’ attention to these images over practice because they became less novel. Therefore, in Experiment 3 we investigated whether stimulus exposure influenced the extent to which participants attended to the stop-associated images. To this end, we halved the number of stimulus presentations in the training phase, such that there were 12 presentations prior to the test phase (cf. 24 presentations in Experiments 1 and 2).

### Method

#### Subjects

Thirty-two students from the University of Exeter participated for monetary compensation (£5; approximately $7.80) or partial course credit (*M* = 19.19 years, *SD* = 1.49, 26 females, 29 right-handed). One participant was excluded because they incorrectly executed a response on ≥30% of stop trials. The data with this participant included are available in the online supplemental material.

#### Apparatus, stimuli, procedure, and analyses

The apparatus, stimuli, and procedure were identical to those of Experiments 1 and 2, except for the following changes: each image was presented once per block (i.e., 14 presentations in total). To ensure that the overall *p*(stop) was the same as in Experiments 1 and 2, the reduced number of image presentations meant that the s–s contingencies for the go and the control images in the test phase had to be altered (for the specific contingencies, see Table 1). As in Experiment 1, the stop-associated images were paired with a stop signal on 75% of presentations during the training phase to provide an index of image-stop learning during training. For comparison with Experiments 1 and 2, in the analyses the blocks were collapsed to ensure that the number of observations per cell was comparable.

### Results

#### Training phase

**Figure 5 fig5:**
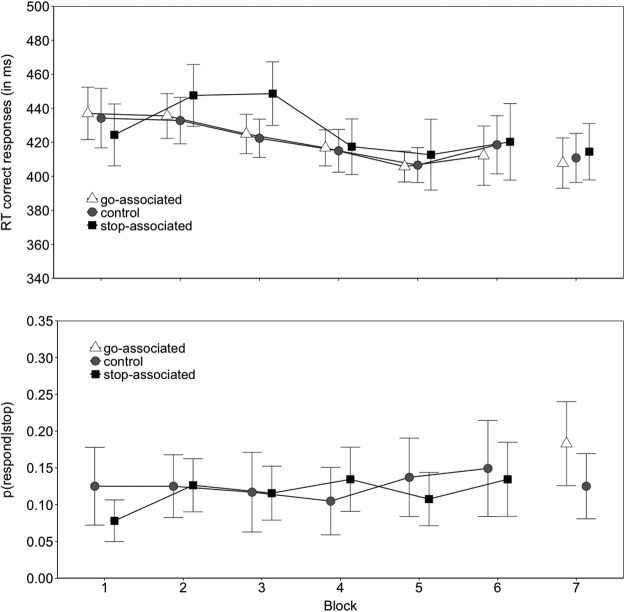
Go reaction times (RTs; upper panel) and *p*(respond|stop) data (lower panel) for the three image types (stop, go, control) as a function of the block (Blocks 1–6 = training phase; Block 7 = test phase) in Experiment 3. Error bars are 95% confidence intervals.

In the training phase, the main effect of image type on go RTs was marginally significant (*p* = .058); planned comparisons revealed marginally significant differences between the stop-associated images (428 ms) and the go-associated images (422 ms), *t*(30) = −1.99, *p* = .055, g_av_ = 0.234, and between the stop-associated images and the control images (422 ms), *t*(30) = −1.92, *p* = .064, g_av_ = 0.242. There was no reliable difference between the control and the go-associated images, *t*(30) = 0.28, *p* = .777, g_av_ = 0.017. However, Figure 5 shows that RTs were longer for the stop-associated images than for the control and the go-associated images in Blocks 1 through 3, but this difference disappeared from Block 4 onward. This conclusion was supported by a reliable interaction between image type and block (*p* = .005). The overall main effect of block was reliable, suggesting that participants improved as a function of task practice (*p* < .001). There were no reliable differences in *p*(respond|stop).[Fig-anchor fig5]

#### Test phase

As in Experiments 1 and 2, there was no main effect of image type on go RT in the test phase (*p* = .479). However, the difference in *p*(respond|stop) between the go-associated images (0.183) and the control images (0.125) was marginally significant, *p* = .062, suggesting that the image-go associations did influence test phase performance to some extent (see Table 4).

#### Expectancy ratings

Consistent with the previous experiments, image type influenced expectancy ratings, *F*(2, 60) = 11.44, *p* < .001, *gen. η^2^* = 0.136. Expectancy ratings were greater for the stop-associated images (5.54) than for the go-associated images (4.57), *t*(30) = −3.50, *p* = .001, g_av_ = 0.850, and the control images (4.76), *t*(30) = −3.44, *p* = .001, g_av_ = 0.687. There was no reliable difference between the control images and the go-associated images, *t*(30) = −1.84, *p* = .075, g_av_ = 0.199. However, the expectancy differences did not correlate with the corresponding RT differences (*r*s ≤ 0.136, *ps* ≥ 0.464).

### Discussion

In Experiment 3, we investigated whether the amount of exposure to the stop-associated images influenced the extent to which the s–s mappings acquired during training affected task performance in the test phase when the s–s mappings were reversed.

Consistent with Experiment 1–2, our results indicate that participants acquired the s–s mappings during training; participants were slower to respond to the stop-associated images than to the go-associated images and the control images. However, this effect appeared and then disappeared again throughout practice; this conclusion was supported by a significant interaction between block and image type. This is consistent with the (numerically) diminished learning effect observed at the end of the training phase in Experiment 1. Furthermore, participants were not slower to respond to the stop-associated images than to the go-associated images and to the control images in the test phase (although we observed a marginally significant difference between go and control images). This suggests that the amount of habituation to the images cannot entirely account for the absence of the test phase effect. This leaves an associability mechanism controlling attention to the stimuli as the most plausible explanation for the results of our experiments so far.

As in Experiments 1 and 2, we find clear evidence that participants acquired the s–s contingencies in the expectancy ratings obtained following task completion; participants expected to stop their response more for the stop-associated images than for the go-associated images and the control images. This suggests that participants did not forget the s–s contingencies, despite the disappearance of the learning effect on task performance toward the end of the training phase and during the test phase.

## Experiment 4

In the final experiment, we presented the image before the go and stop signals, and asked participants to rate whether they expected to stop or not. Furthermore, we presented the go and stop signals around the image, at one of four possible locations (one of four corners of the image; for a similar procedure see Houben & Jansen, 2011). These manipulations served two purposes. First, the results of Experiments 1 through 3 suggested that participants stopped paying attention to the task-irrelevant images. We tried to increase attention to the images by making them perfect predictors of the outcome (Experiment 2) or by decreasing image habituation (Experiment 3). These manipulations were only moderately effective: some behavioral indices indicate that our manipulation influenced learning, but the effect of learning on test performance still disappeared over training. By presenting the images before the go and stop signals, and asking participants to rate their stop expectancy, participants were less likely to ignore the images in Experiment 4 (however, subjects were not explicitly instructed to attend to the images so as to keep the image-stop mappings implicit as in Experiments 1 through 3). Furthermore, the images initially did not have the stop signal present as a competitor driving their associability down. If our attentional account is correct, we should observe the effects of stop training in the later blocks of the training phase and in the test phase. Second, in Experiments 1 through 3, we found that participants generated expectancies based on the image-stop associations acquired during training. In Experiment 1, expectancy correlated with some aspects of performance in the test phase, but we could not replicate this finding in Experiments 2 through 3. It is possible that obtaining the expectancy ratings following task performance meant that these expectancies were contaminated by the relearning of the new (inconsistent) mappings in the test phase. Therefore, in Experiment 4, we further investigated the role of expectancy in s–s learning by obtaining expectancy ratings during task performance (for a similar procedure, see, e.g., McAndrew, Jones, McLaren, & McLaren, 2012; Perruchet, Cleeremans, & Destrebecqz, 2006).

### Method

#### Subjects

Thirty-two students from the University of Exeter participated for partial course credit (*M* = 18.47 years, *SD* = 0.62 years, 27 females, 31 right-handed). Four participants were excluded because they incorrectly executed a response on ≥30% of stop trials. The data with these participants included are available in the online supplemental material.

#### Apparatus, stimuli, procedure, and analyses

The apparatus, stimuli, and procedure were identical to those of Experiment 3, except for the following changes: all trials began with the presentation of the image in the center of the screen. The word *RATING* was presented above and below the image to instruct participants to rate “How much do you expect to withhold your response?” Participants inputted their ratings on a scale, ranging from 1 (*I definitely do not think that I will have to withhold my response*) to 9 (*I definitely think that I will have to withhold my response*) using the number keys of the keyboard with their right index finger (latency rating response: *M* = 969 ms; *SD* = 681 ms). After participants made their expectancy rating, a go/stop signal appeared at one of four locations on the screen (top-left, bottom-left, top-right, or bottom-right corner of the image). The delay between the expectancy response and the presentation of the go/stop signals varied randomly between 500 and 1,250 ms. Participants responded on go trials by pressing the spacebar on a keyboard with their left index finger. To allow for the presentation of the signals at each location on the screen, task rules used in Experiments 1 through 3 that were based on signal location (e.g., X on the left/right of the image) or signal shape (e.g., shape bigger/smaller than a 50 pence piece) were excluded and, of the remaining rules, seven rules were selected on the basis of response latencies in Experiments 1 through 3 using a nonparametric box and whisker method (Tukey, 1977). A full list of the signals used appears in Appendix A. The expectancy ratings data in the training and test phase trials were analyzed separately using ANOVAs with image type and block as within-subjects factors.

### Results

#### Training phase

**Figure 6 fig6:**
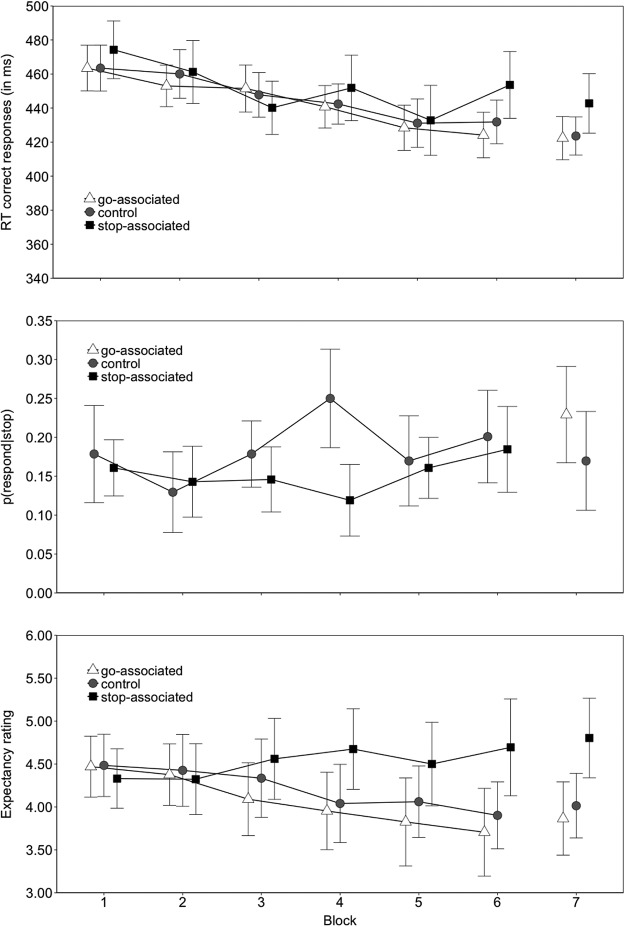
Go reaction times (RTs; upper panel), *p*(respond|stop) data (middle panel) and expectancy ratings (lower panel) for the three image types (stop, go, control) as a function of the block (Blocks 1–6 = training phase; Block 7 = test phase) in Experiment 4. Error bars are 95% confidence intervals.

In the training phase, there was a reliable interaction between image type and block on go RTs (*p* = .03), reflecting slower responding for the stop-associated images than for the go-associated images and the control images in the second half of the training phase (see Figure 6). The *p*(respond|stop) was also lower for the stop-associated images (0.152) than for the control images (0.185) (*p* = .011). The interaction between image type and block in the *p*(respond|stop) was not reliable. [Fig-anchor fig6]

The analysis of the online expectancy ratings also revealed a reliable image type by block interaction (*p* = .005), reflecting higher stopping expectancies for the stop-associated images in the second half of the training phase (Blocks 4–6; see Figure 6). There was also a reliable main effect of block on the expectancy ratings (*p* = .012): overall mean expectancy ratings decreased with task practice, which is consistent with the overall *p*(stop) of 0.25 (note, the increase in expectancy ratings across block for the stop-associated images was not reliable, *p* = .261). Combined, these findings indicate that participants were generating appropriate expectancies during the acquisition of the s–s mappings. Importantly, the overall stop-minus-go expectancy ratings difference reliably correlated with the corresponding RT difference in the training phase, *r*(26) = 0.575, *p* = .001; the overall stop-minus-control expectancy ratings difference also correlated with the corresponding RT difference, *r*(26) = 0.498, *p* = .006.

#### Test phase

Unlike in Experiments 1 through 3, we found a main effect of image type on go RTs in the test phase (*p* = .004). Planned comparisons revealed that responding to the old stop-associated images was slower (443 ms) than to the go-associated images (422 ms), *t*(27) = −2.84, *p* = .008, g_av_ = 0.517, and to the control images (424 ms), *t*(27) = −2.87, *p* = .007, g_av_ = 0.542. There was no reliable difference between the go and control images, *t*(27) = −0.31, *p* = .756, g_av_ = 0.040, (Figure 6; Table 3). Image type did not reliably influence *p*(respond|stop) in the test phase (however, the means were in the predicted direction, see Figure 6; Table 4).

There was also a reliable main effect of image type on test phase expectancies (*p* = .002); planned comparisons revealed that participants expected to stop more for the old stop-associated images (4.80) than for the go-associated images (3.86), *t*(27) = −2.65, *p* = .013, g_av_ = 0.807, and the control images (4.01), *t*(27) = −2.83, *p* = .008, g_av_ = 0.719. There was no reliable difference between the go-associated and the control images, *t*(27) = −1.37, *p* = .181, g_av_ = 0.143. As in the training phase, we found that the stop-minus-go expectancy ratings difference reliably correlated with the corresponding RT difference, *r*(26) = 0.624, *p* < .001; the stop-minus-control expectancy ratings difference also correlated with the corresponding RT difference, *r*(26) = 0.653, *p* < .001. Hence, participants who had a stronger expectancy to stop their response when the stop-associated images were presented displayed greater response slowing for these images than for the go-associated images and for the control images upon signal presentation.

To further investigate to what extent the expectancy to stop determined response slowing for the stop-associated images, we conducted a median-split analysis on the expectancy ratings of the test phase (we could not perform a similar analysis in the training phase because there were not enough trials in each block). We calculated the median for each image type and participant separately. Ratings greater than the median were classified as a stop expectancy, whereas ratings less than or equal to the median were classified as a go expectancy. Four participants were excluded from these analyses as they always entered the same expectancy rating for one or more of the image types (consequently, we could not perform a median split). We analyzed the data with a 2 (expectancy: stop vs. go) × 3 (image type) ANOVA. Consistent with previous work on proactive control (see, e.g., Verbruggen & Logan, 2009), responding was slower for trials on which participants expected a stop signal (445 ms) compared with trials on which participants expected a go signal (420 ms), *F*(1, 23) = 13.96, *p* = .001, *gen. η^2^* = .088. As discussed above, image type also had a reliable main effect on performance. Importantly, the effects of s–s learning and expectancy were additive; that is, the two-way interaction between expectancy and image type was not reliable, *F*(2, 46) = .08, *p* = .915, gen. η^2^ < .001 (for descriptive statistics, see Table 5). Thus, the slowing for the stop-associated images is unlikely to reflect an entirely strategic, expectancy-driven effect.[Table-anchor tbl5]

### Discussion

Consistent with the results of Experiments 1 through 3, we find evidence that participants acquired the s–s associations. In the training phase, responding became slower for the stop-associated images than for the go-associated images and the control images with task practice, and the *p*(respond|stop) was lower for the stop-associated images than for the control images. In addition, the expectancy ratings showed that participants generated expectancies that were consistent with the trained s–s contingencies in the second half of the training phase. These expectancies correlated with task performance in the training phase: participants who expected to withhold their response more to the stop-associated images responded more slowly to these images than to the go-associated images and to the control images during training. Unlike in Experiments 1 through 3, we find that learning also influenced performance in the test phase: participants were slower to respond to the stop-associated images than to the go-associated images and the control images during the test phase.

Our results suggest that presenting the images before the go/stop signals and asking participants to rate their expectancy on each trial increased the extent to which participants attended to these images. To ensure that attention to the task-irrelevant images was maximized, we combined these manipulations in the same procedure. As a consequence, we cannot determine the relative contributions of these manipulations to the observed slowing for the stop-associated images in the test phase. One could speculate that the observed slowing reflects an entirely strategic, expectancy-driven effect, rather than the implicit retrieval of the acquired s–s associations (as predicted by the automatic inhibition account). We argue that this explanation is unlikely for several reasons. First, our median split analysis on expectancy ratings in the test phase shows that the slowing for the stop-associated images occurred even when stop signal expectancy was relatively low. This result suggests that expectancy ratings cannot account for the whole data pattern.^3^ Second, previous studies have demonstrated stop-learning effects using procedures in which the stop-associated stimuli are presented prior to stop-signal onset but, unlike the present experiment, without expectancy ratings on each trial. For example, in a recent study we presented the stop-associated stimuli as warning cues for a variable duration prior to the presentation of the stop signal, and observed stop learning effects during the training and test phases (Bowditch, Verbruggen, & McLaren, 2015). Similarly, Veling and colleagues have conducted two experiments using go/no-go designs in which food images were presented 100 ms (Veling, van Koningsbruggen, Aarts, & Stroebe, 2014) or 500 ms (Veling, Aarts, & Stoebe, 2013) prior to the onset of the go/no-go signal. They found that when the food images were consistently presented on no-go trials, subsequent choice of the food items was reduced (Veling, Aarts, & Stroebe, 2013) and weight loss was facilitated (Veling et al., 2014). Finally, research in the wider action control literature is consistent with the pattern of findings in the present study. For example, Frings and Moeller (2012) found that associations between old distractor stimuli and the previously required target response only interfered with responding when the distractors were presented prior to the target stimuli. Combined, these studies suggest that presenting the task-irrelevant image before the go or no-go signal increases attention to the images, and consequently, the probability that the image-stop associations are retrieved. However, future research is required to determine the relative contributions of increased attention and expectancies (see, e.g., Best, Stevens, McLaren, & Verbruggen, 2015; see Footnote 3).

To conclude, the presence of a learning effect in the test phase is consistent with our hypothesis that attention to the images determines whether acquired s–s associations influence behavior in the test phase. Now that the images are task-relevant and associability is no longer driven down for the images by virtue of their competition for attention with the stop signal, we see a strong effect on test phase go RTs. Furthermore, the test-phase expectancy ratings show that participants continued to generate expectancies consistent with the image-stop mappings acquired during training, despite the reversal of these mappings. As in the training phase, these expectancies reliably correlated with task performance: participants who expected to withhold their response more for the stop-associated images responded more slowly to these images than to the go-associated images and to the control images in the test phase. However, the median split also suggested a contribution of implicit (nonexpectancy related) processes.

## General Discussion

In the present study, we investigated three outstanding issues relating to the mechanisms of s–s learning. The first two issues were highlighted in our recent review on s–s learning (Verbruggen, Best, et al., 2014): (1) are associations between stimuli and stopping direct, and (2) what is the role of expectancy in s–s learning? On the basis of the results of Experiment 1, Experiments 2 through 4 also investigated a third issue: (3) does attention to the stop items affect the extent to which s–s learning influences behavior? On the basis of our findings, we can answer each of these questions.

### Are Associations Between Stimuli and Stopping Direct?

Across four experiments where the specific stop signals and rules were always changing, we provide strong evidence for the idea that participants can learn direct s–s associations (Verbruggen & Logan, 2008a). During training, we found that responding was slower (Experiments 1, 3, and 4; in Experiment 2, we could not compare stop- and go-associated images in the training phase) and the *p*(respond|stop) was lower (Experiment 1 and Experiment 4) for images that were consistently associated with stopping than for images associated with going and for control images that were not particularly associated with stopping or going.

In recent experiments, we have observed that learning can influence the *p*(respond|stop) but not response latencies on go trials (see, e.g., Experiment 2 in Verbruggen, Best, et al., 2014). On the basis of previous findings in the conditioning literature (for a review, see Hall, 2002), we hypothesized that participants in these experiments learned an association between an item and a representation of a no-go or stop signal. Hence, when the item was repeated, it primed the signal so that it was detected sooner on stop-signal trials, resulting in improved response inhibition and, consequently, a lower *p*(respond|stop). The signal priming idea explains why it can be that learning influences the probability of stopping on stop-signal trials without influencing response latencies on go trials. In the present study, both RTs and *p*(respond|stop) were influenced even though the go/stop signals and task rules constantly changed (and there were two go signals and two stop signals in each block). This indicates that learning was not (solely) mediated via image-signal associations. The most parsimonious account is that the effects in the present study reflect the direct association of the stop-associated images with a stop goal rather than the association of the stop-associated images with the representation of a single stop signal. Therefore, the present study provides the strongest evidence to date for the original automatic inhibition hypothesis of s–s (goal) learning. In situations in which the task rules do not constantly change, it is likely that individuals will acquire both stimulus-goal and stimulus-signal associations (indeed, research in the conditioning literature suggests that the acquisition of multiple associations is the norm; Hall, 2002). It is possible that experimental factors, such as the perceptual properties of the stop signal, will influence which association dominates behavior.

It is important to note that the learning effects demonstrated in the present study are assumed to reflect the acquisition of s–s associations rather than the absence of stimulus–go learning on stop trials. Although the “absence of go learning” explanation may initially seem parsimonious, it cannot account for several findings previously reported in the stop-learning literature. First, we have previously demonstrated that responding to old stop items is slowed compared with novel items that were not presented during training (hence, these items were not associated with going or stopping; Verbruggen & Logan, 2008a, Experiment 1). Second, neuroimaging work has shown that the presentation of old stop items activates the neural inhibitory control network (Lenartowicz et al., 2011; but see also below). Third, brain stimulation studies have shown that even when the probability of go and no-go signals is equal (i.e., 50/50), motor-evoked potentials are below baseline 200 ms to 300 ms following no-go stimulus presentation (indicating that responding is suppressed; Leocani, Cohen, Wassermann, Ikoma, & Hallet, 2000). In other words, successful performance on a no-go trial requires the activation of a no-go or stop response and not just the absence of a go response. Fourth, short-term aftereffects of stopping further support the idea that participants can learn s–s associations that can have a (global) inhibitory effect on responding (Giesen & Rothermund, 2014). Finally, in the present experiments, response latencies decrease for go and control images but we observe an initial increase in response latencies for stop-associated images over practice (Experiment 1). In Experiments 3 and 4, this conclusion is further supported by a reliable interaction between image type and block. Finally, the comparison of expectancy ratings in Experiments 1 and 2 revealed that expectancy ratings were altered when the image-stop consistencies had changed (even though the image-go contingencies did not change). Therefore, previous results and the findings reported in the present study are consistent with the idea that participants can learn go associations on go trials and stop associations on stop trials (which interfere with responding).

### What Is the Role of Expectancy in s–s Learning?

In the present study, we show that participants generated expectancies that were consistent with the s–s mappings acquired during training: participants expected to withhold their responses more when stop-associated images were presented than when go-associated and control images were presented. Furthermore, these expectancy ratings were sensitive to the specific contingencies in play: participants expected to withhold their responses more for the stop-associated images that were reinforced on 100% of presentations (Experiment 2) than for the stop-associated images that were reinforced on 75% of presentations (Experiment 1). Finally, we found that these expectancies correlated with task performance both during the acquisition of the s–s mappings in the training phase (Experiment 4) and following the reversal of these mappings in the test phase (Experiment 1 and Experiment 4).

The role of expectancies in s–s learning has not been previously investigated. Therefore, the present study provides the first evidence that s–s learning is partly mediated via explicit knowledge of the s–s contingencies in play (although the median split analysis and the absence of significant correlations in some of the experiments indicate that implicit processes must play a role as well). This could indicate that the response slowing observed for the stop-associated images is caused by top-down control processes. First, the slowing could be partly due to proactive control. According to this proactive control account of s–s learning, stop items could become predictive cues (e.g., if image X then *p*(stop) is high) that indicate that participants should adjust their response strategies accordingly. If this were the case, this would suggest that earlier findings that have demonstrated response slowing and neural activation of the inhibitory control network by old stop items (Lenartowicz et al., 2011) could be due to proactive control (i.e., another algorithmic process), rather than the direct activation of the stop response via memory-retrieval (i.e., picture X = stop). Therefore, though the retrieval of the s–s association may still be automatic, the subsequent slowing observed following the reversal of the s–s mapping would be due to a top-down control process (rather than a bottom-up process as is currently assumed). Second, stop items could effectively become a new stop signal (the direct stopping account). In other words, the only difference between the stop items and an external stop signal is that the association with stopping is acquired via learning in the case of the stop items, whereas it is acquired via instructions in the case of the stop signal. Thus, in both cases, response inhibition is a deliberate act of control. But the advantage of the former form of control is that the go and stop processes in stop-signal tasks could be initiated simultaneously and, therefore, start the race at the same time (Logan & Cowan, 1984); consequently, response inhibition is more likely to succeed.

It is important to note, however, that the proactive control route and the direct stopping route are both compatible with the idea that associative learning plays a key role in response inhibition paradigms; indeed, both accounts still assume that stimulus-specific learning influences stop performance. Learning offers participants another route to control their behavior. The key difference between these two top-down accounts and the ‘automatic’ inhibition account is the nature of the process that occurs following the retrieval of the s–s association; either this association directly activates the stop goal via an s–r-based link (in the automatic stopping account) or this association indirectly activates the stop goal via a top-down (algorithmic and deliberate) control process. Future research is required to distinguish between these accounts (see, e.g., Best, Stevens, et al., 2015).

### Does Attention to the Stop Items Affect the Extent to Which s–s Learning Influences Behavior?

In Experiments 1 through 3, the acquired s–s associations did not influence performance in the test phase, despite effects of learning on task performance in the training phase and on expectancies following task completion (suggesting that participants had not forgotten the s–s associations).

A potential explanation for this finding is that the images used in the present study were task-irrelevant so participants may have begun to ignore the images as they became less novel and as they learned that they were less predictive. In Experiments 1 through 3, task performance did not require participants to attend to the stop-associated stimuli (unlike in our previous work; see, e.g., Verbruggen & Logan, 2008a), so participants may have started ignoring all the images over time. In line with this possibility, the effect of image type reliably interacted with block (in Experiment 3) and visual inspection of the data shows that the influence of image-stop learning on performance began to disappear at the end of the training phase (Experiment 1). Because there were no differences between the image types in the final block of the training phase, this may explain why we did not find any effect of image-stop learning in the test phase.^4^ Several associative learning accounts suggest that the reduced predictiveness of the images relative to the go/stop signals (in Experiments 1 and 3) may have decreased the extent to which they were considered informative or salient and, consequently, the extent to which participants attended to them and the extent to which they can influence performance (Mackintosh, 1975). Effects that point to this conclusion have been previously observed in animals (see, e.g., Sutherland & Mackintosh, 1971 for a review of this literature) and, it should be noted, in humans (Le Pelley & McLaren, 2003; Livesey & McLaren, 2007; Suret & McLaren, 2005). For example, Le Pelley and McLaren (2003) showed that foods that were worse predictors of an outcome than other foods present on a trial in an allergy discrimination task became less salient, resulting in slower learning of a new association to these stimuli in a later training phase (cf. learned irrelevance; Mackintosh, 1975). Note that the majority of our results in Experiments 1 through 3 are also consistent with conflict monitoring accounts (e.g., Botvinick et al., 2001), which predict that participants will ignore task-irrelevant information that produces response conflict or choice errors. However, unlike the associative learning accounts, these conflict monitoring accounts do not easily explain the absence of a learning effect in the test phase found in Experiment 2 when conflict should have been minimized by the use of 100% contingencies.

It is possible that the use of neutral images increased the extent to which participants began to “tune out” their attention. Motivationally salient images capture attention even if they are task-irrelevant (e.g., Anderson, Laurent, & Yantis, 2011). Consequently, if task-irrelevant, but motivationally salient images are used as the stop-associated stimuli, the attentional capture to the images would be increased, and the “tuning out” of attention could be slowed. Thus, the salience of task-irrelevant stop-associated stimuli could be a key consideration for applied studies examining the effects of no-go training effects on food and alcohol consumption.

It should be noted that we found a clear effect of s–s learning on test phase performance when attention to the images was increased in Experiment 4 (as a result of presenting the images before the go/stop signals and the requirement to make an online expectancy rating on each trial). This finding is consistent with the instance theory (Logan, 1988; Logan & Etherton, 1994) and other theories of associative learning. For example, instance theory suggests that processing episodes will only be stored and retrieved from memory when participants attend to each stimulus presentation (Logan, 1988; Logan & Etherton, 1994). Thus, by encouraging subjects to attend to the image in Experiment 4, the image-stop associations were more likely to be retrieved, and performance was influenced in the test phase. Therefore, the present study strongly indicates that the influence of image-stop learning on behavior is likely to be determined by the interplay of both attentional control and associative learning systems (see also Logan, 1988; Verbruggen, McLaren, & Chambers, 2014).

### Wider Implications

In addition to contributing to our theoretical understanding of s–s learning, the present study has implications for more applied research. First, our results indicate that attentional settings influence learning in response inhibition tasks. Even when salient images are used as stimuli (e.g., as in the food studies mentioned above), participants may still adjust their attentional settings, and ignore the images to a certain degree. Currently, the task-relevance of the stop-associated images used in stop-training studies varies. Although the task-relevance of the images may not influence engagement in impulsive behaviors (e.g., impulsive eating can be prompted by implicit processing of food cues in the environment), our results suggest that designs in which participants must attend to the images should produce stronger s–s associations that will have a more pronounced influence on stop learning. Second, the present study indicates that it is possible to learn a direct association between a stimulus and a stop goal or the act of withholding a response when multiple signals are used. When only one signal is used, there is the possibility that participants will learn stimulus-signal associations (as our recent results suggest; see above). Thus, if the aim is to obtain inhibition training effects that transfer to real-world settings where stop signals are no longer present, multiple signals may be preferable.

To maximize the inhibitory control training effects, we thought it important to consider other features of the stop learning task. In the present study, we devised a novel task that combined features of the go/no-go task and the stop-signal task. In Experiments 1 through 3, the delay between the presentation of the images and the go/stop signal was 0 ms; in Experiment 4, the go and stop signal also occurred at the same moment (i.e., there was no delay between the go and the stop signal). But to avoid that subjects would simply wait on all trials, we used a low overall proportion of stop trials (.25), imposed a relatively strict response deadline (750 ms) and provided feedback if the participant did not respond in time. We believe that this hybrid design is optimal to investigate stop learning it allows us to manipulate the go/stop signal representation while maximizing the number of correct stop trials. After all, our previous work indicates that s–s associations are less likely to be learned when inhibition is unsuccessful (Verbruggen & Logan, 2008a, 2008b; Verbruggen et al., 2008). The idea that the stop outcome is important is further supported by studies in the applied domain. Stop learning effects on task performance and on food and alcohol consumption have been observed after both go/no-go and stop-signal training (see preceding paragraphs). However, a recent meta-analysis indicates that go/no-go training has stronger effects on appetitive behavior than stop training (Jones et al., 2015). This could be due to generally higher success rates in the go/no-go task (Jones et al., 2015; Verbruggen & Logan, 2008a). Neuroimaging research also shows that, despite some overlap, there may be several differences in the neural substrates of the go/no-go and stop-signal tasks (for a discussion; see, e.g., Eagle, Bari, & Robbins, 2008; Swick, Ashley, & Turken, 2011). Thus, it is possible that the differences between the training protocols could be due to other factors as well. Future research is required to investigate the specific action control processes influenced by stop learning in these tasks.

Finally, our results indicate that expectancies also play a role in stop-learning paradigms. It is possible that differences in the expectancy to stop are present in applied studies, especially as the go/stop rule is typically simpler (and remains the same throughout the task), the image-stop mappings are more explicit, and the stimulus set smaller than in the present study. In applied studies, expectancies and demand characteristics may play an important role (Boot et al., 2013). However, it is currently unclear the extent to which the expectancy effects observed in the present study relate to the demand characteristics identified by Boot and colleagues (2013) and, indeed, the behavioral findings of applied stop-training studies. For example, there are some procedural differences between the present study and stop-training studies (e.g., in Experiment 4, we obtained an expectancy rating on every trial). Similarly, though our results show a relationship between expectancy and go RT, it is unclear the extent to which expectancy equates to other dependent variables used in the stop-training studies, such as food intake or stimulus devaluation (see, e.g., Wessel, O’Doherty, Berkebile, Linderman, & Aron, 2014). For example, a recent stop-training study from our lab suggests that though a substantial proportion of participants became aware of the s–s contingencies during training (in a funneled debrief, 83% of participants in Experiment 1 and 74% of participants in Experiment 2 reported knowledge that specific images were associated with stopping), the majority of participants did not expect these image-stop associations to influence their subsequent food intake (Lawrence et al., 2015). Nevertheless, we believe that future applied research should include a dependent variable that is sensitive enough (see Newell & Shanks, 2014), such as expectancy ratings (e.g., Stothart, Simons, Boot, & Kramer, 2014), to examine the extent to which the behavioral effects observed both during and following inhibitory control training relate to the expectancy to stop.

### Conclusion

In sum, the present findings indicate that participants can learn direct associations between stimuli and a stop goal when the go/stop rule changes at the beginning of each block. Exposure to the image-stop associations influenced task performance during training, and expectancies following task completion. However these results also suggest that attention to stimulus attributes is key for the retrieval of processing episodes; if participants do not attend to the stop-associated stimulus then the previously acquired s–s associations will not influence behavior. Our results are consistent with the instance theory and other attentional accounts of associative learning.

## Supplementary Material

10.1037/xhp0000116.supp

## Figures and Tables

**Table 1 tbl1:** Proportion of Stop-Signal Trials as a Function of Experiment, Image Type, and Phase

Experiment/Image type	No. of images	Percentage stop-signal trials	
		Training phase	Test phase
Experiment 1			
Stop-associated	8	75	0
Go-associated	16	0	8 images: 75; 8 images: 0
Control	16	25	25
Experiment 2			
Stop-associated	10	100	0
Go-associated	30	0	20 images: 0; 10 images: 100
Experiment 3 and Experiment 4			
Stop-associated	8	75	0
Go-associated	16	0	4 images: 0; 12 images: 50
Control	16	25	8 images: 0; 8 images: 50
*Note.* The overall *p*(stop-signal) across experiments and within the experimental phases was .25.			

**Table 2 tbl2:** Probability of a Missed Go Response (p[Miss]) as a Function of Experiment, Image Type, and Experimental Phase

Experiment/Image type	Training phase		Test phase	
	*M*	*SD*	*M*	*SD*
Experiment 1				
Stop-associated	.020	.071	.013	.033
Go-associated	.015	.024	.014	.024
Control	.016	.028	.017	.036
Experiment 2				
Stop-associated			.024	.043
Go-associated	.016	.025	.013	.031
Experiment 3				
Stop-associated	.028	.083	.038	.060
Go-associated	.023	.034	.037	.062
Control	.018	.031	.036	.052
Experiment 4				
Stop-associated	.028	.088	.018	.033
Go-associated	.020	.032	.007	.018
Control	.021	.032	.025	.036
*Note.* *P*(miss) is the ratio of the number of omitted responses to the total number of no-stop-signal trials: *p*(miss) = missed/(correct + missed).				

**Table 3 tbl3:** Overview of Repeated Analyses of Variance Performed to Compare Go and Stop Training Phase Performance

Experiment/factor	*df* = 1	*df* = 2	SS1	SS2	*F*	*p*	η^2^
Experiment 1							
Go reaction time							
Image type	2	56	21,980	41,878	14.70	<.001	.009
Block	11	308	43,427	143,8376	.85	.575	.017
Image type × Block	22	616	17,475	431,981	1.13	.331	.007
*p*(respond|stop)							
Image type	1	28	.071	.323	6.17	.019	.005
Block	11	308	.547	7.616	2.01	.043	.040
Image type × Block	11	308	.154	3.384	1.28	.238	.011
Experiment 2							
Go reaction time							
Block	11	319	27502	405,062	1.97	.039	.037
*p*(respond|stop)							
Block	11	319	.199	3.846	1.50	.136	.037
Experiment 3							
Go reaction time							
Image type	2	60	5589	47,836	3.51	.058	.006
Block	5	150	64257	437,275	4.41	<.001	.061
Image type × Block	10	300	16048	162,531	2.96	.005	.016
*p*(respond|stop)							
Image type	1	30	.010	.200	1.48	.232	.002
Block	5	150	.053	2.688	.60	.703	.009
Image type × Block	5	150	.055	1.780	.93	.461	.009
Experiment 4							
Go reaction time							
Image type	1	27	6447	70,581	2.47	.128	.012
Block	5	135	51108	157,194	8.78	.000	.085
Image type × Block	5	135	12088	128,773	2.53	.037	.021
*p*(respond|stop)							
Image type	1	27	.087	.317	7.45	.011	.015
Block	5	135	.109	1.857	1.58	.173	.019
Image type × Block	5	135	.180	2.363	2.05	.077	.031
*Note.* Image type (Experiments 1, 3, and 4: stop-associated, go-associated, control) and block (Experiments 1 and 2: 1–12; Experiments 3 and 4: 1–6) are the within-subjects factors. We did not analyze *p*(miss) because values were low. SS = sum of squares.							

**Table 4 tbl4:** Overview of Repeated Analyses of Variance Performed to Compare Go and Stop Test Phase Performance

Experiment/factor	*df* = 1	*df* = 2	SS1	SS2	*F*	*p*	η^2^
Experiment 1							
Go reaction time							
Image type	2	56	471	22425	.59	.557	.002
Block	1	28	2318	53598	1.21	.281	.010
Image type × Block	2	56	771	13040	1.66	.200	.003
*p*(respond|stop)							
Image type	1	28	.001	.268	.16	.695	<.001
Block	1	28	.058	.380	4.24	.048	.028
Image type × Block	1	28	.007	.316	.64	.429	.004
Experiment 2							
Go reaction time							
Image type	1	29	160	10621	.44	.513	<.001
Block	1	29	390	47352	.24	.629	.002
Image type × Block	1	29	60	7087	.25	.624	<.001
*p*(respond|stop)							
Block	1	29	.020	.337	1.73	.198	.019
Experiment 3							
Go reaction time							
Image type	2	60	711	28880	.74	.479	.005
*p*(respond|stop)							
Image type	1	30	.51	.416	3.73	.062	.043
Experiment 4							
Go reaction time							
Image type	2	54	7329	28228	7.01	.004	.064
*p*(respond|stop)							
Image type	1	27	.050	.473	2.83	.104	.034
*Note.* Image type (Experiments 1, 3, and 4: stop-associated, go-associated, control, Experiment 2: stop-associated, go-associated) and block (Experiments 1 and 2: 13–14; Experiments 3 and 4: 7) are the within-subjects factors. We did not analyze *p*(miss) because values were low. SS = sum of squares.							

**Table 5 tbl5:** Go Reaction Times (in ms) in the Test Phase as a Function of Expectancy (Go, Stop) and Image Type (Stop-Associated, Go-Associated, Control) in Experiment 4

	Stop expectancy		Go expectancy	
Image type	*M*	*SD*	*M*	*SD*
Stop-associated	453	56	429	51
Go-associated	437	43	411	36
Control	440	40	411	33

**Table B1 tbl6:** Means and Standard Deviations of Go Reaction Times (RTs) and Probability of Responding on Stop Trials (p(R|S) for Each Block

Measure	1	2	3	4	5	6	7	8	9	10	11	12	13	14	15	16	17	18	19	20	21	22
Go RT																						
*M*	341	324	327	329	322	323	331	322	324	325	324	319	325	322	316	318	**317**	**323**	**316**	**325**	**321**	**311**
*SD*	31	25	25	35	33	33	36	32	33	33	29	34	35	39	36	33	**34**	**37**	**35**	**31**	**36**	**31**
*p*(R|S)																						
*M*	.03	.01	.02	.01	.01	.02	.03	.01	.02	.01	.01	.03	.03	.01	.01	.02	**.02**	**.03**	**.03**	**.02**	**.01**	**.02**
*SD*	.05	.02	.03	.03	.02	.03	.05	.02	.05	.03	.03	.04	.05	.03	.03	.03	**.04**	**.04**	**.04**	**.03**	**.03**	**.03**
*Note.* Test blocks are in boldface.																						

## Footnotes

1 In this context, a *task goal* refers to an abstract representation of going or stopping; in other words, it does not specify which specific response or motor program has to be executed. Consistent with the goal account, Giesen and Rothermund (2014) recently demonstrated that stop associations may have global effects on responding.

2 There is some evidence that suggests that inconsistent reinforcement can increase attention to, and motivation salience of, conditioned stimuli. For example, the Pearce & Hall (1980) model suggests that associability is maintained for stimuli that are followed by unpredictable outcomes. However, despite animal data in support of this effect (e.g., Anselme, Robinson, & Berridge, 2013), there is relatively little data showing this effect in humans (see Hogarth, Dickinson, Austin, Brown, & Duka, 2008). The weight of evidence using humans participants is in favor of the Mackintosh (1975) model outlined in preceding text (but for a combination of both algorithms in one model, see Pearce & Mackintosh, 2010).

3 This conclusion is further supported by a recent study in which we directly manipulated knowledge of the s–s contingencies while measuring attentional focus during task performance (Best, Stevens, et al., 2015). The data pattern in the explicit condition was similar to the pattern observed in a proactive control study using the same paradigm (Verbruggen, Stevens, et al., 2014). The data pattern looked qualitatively different in the implicit condition, suggesting that the response slowing for stop-associated items was not (entirely) because of proactive control adjustments resulting from the expectancy to stop.

4 We can rule out the possibility that the absence of a test phase effect in Experiments 1 through 3 is due to the use of images or the frequent rule switching. In Appendix B, we present the results of an experiment in which we used a word version of the go/stop task with a single rule. In this experiment, reversing the word-go/stop mapping in the test phase did not influence performance either.

## A A Full List of the Rules and the Stimuli Used in Experiments 1–4

In Experiments 1 through 3, we used 14 go/stop rules. In Experiment 4, we used Rules 1 through 7 only. The signals used in Rules 1 through 11 were presented in Arial font (size = 50). The sizes of the signals used in Rules 12 through 14 are provided in pixels below (screen resolution: 1680 × 1050)*.*

1 Vowels (*a* or *e*) versus consonants (*t* or *n*).
2 Symbols that are the same (@@ or &&) versus symbols that are different (@& or &@).
3 Uppercase letters (*H* or *R*) versus lowercase letters (*h* or *r*).
4 Long symbol strings (£%£% or %£%£) versus short symbol strings (£% or %£).
5 Curved letters (*S* or *C*) versus angled letters (*K* or *W*).
6 Digits smaller than 5 (2 or 4) versus digits bigger than 5 (6 or 8).
7 Curly brackets ({ or }) versus square brackets ([ or ]).
8 Words that refer to animals (*horse* or *sheep*) versus words that refer to fruit (*lemon* or *apple*).
9 Symmetric letter strings (*UYYU* or *YUUY*) versus asymmetric letter strings (*YYUU* or *UUYY*).
10 Crosses on the left of image versus crosses on the right of the image relative to the center (crosses appeared at the top and bottom of the image).
11 Asterisks on the top of the image versus asterisks on the bottom of the image relative to the center (asterisks appeared on the left and right of the image).
12 Horizontal lines (lines appeared across the top or bottom of the image relative to the center; width: 240 pixels) versus vertical lines (lines appeared along the left or right of the image relative to the center; height: 240 pixels).
13 Shapes bigger than a 50 pence piece (square or circle; 100 × 100 pixels) versus shapes smaller than a 50 pence piece (square or circle; 40 × 40 pixels).
14 Lines thicker than a matchstick versus lines thinner than a matchstick (lines appeared horizontally [width = 240 pixels] or vertically [height = 240 pixels] about the center of the image).

## B A Word Version of the Go/Stop Task With a Single Rule

We also ran a word version of the go/stop task. On each trial, a colored word was presented. Half of the participants were instructed to respond when the ink color was green (in other words, green was the go signal), but to refrain from responding when the ink color was red (in other words, red was the stop signal). The color-go/stop mapping was reversed for the other participants (i.e., red = go; green = stop). In the training phase, half of the words always occurred on go trials; the other words always occurred on stop trials. In the test phase, the word-go/stop mapping was reversed.

### Method

#### Subjects

Twenty subjects from Vanderbilt University participated for monetary compensation ($12). All subjects reported normal or corrected-to-normal vision and were native speakers of English.

#### Apparatus and Stimuli

The experiment was run on a PC running Tscope (Stevens, Lammertyn, Verbruggen & Vandierendonck, 2006) and the stimuli were presented on a 21-in monitor. A list of 32 words was drawn from a list of 640 words used by Arrington and Logan (2004): *bamboo, barn, bead, blackboard, boar, buffalo, cabin candle, cattle, cup, elephant, elm, eucalyptus, ferret, grasshopper, hammer, hamster, holly, leash, lemon, mushroom, paddle, rose, scissors, shamrock, shoelace, ski, slug, suitcase, toothbrush, tripod, trombone*. For every subject, two random subsets of 16 words were selected. The first subset was presented on go trials in the training phase and on stop trials in the test phase; the second subset was presented on stop trials in the training phase and on go trials in the test phase. All stimuli were presented in a white lower case Courier font on a black background and ranged from 12 to 52 mm in width (approximately 1.1° to 5.0°) and 4 to 7 mm (approximately 0.4° to 0.7°) in height.

#### Procedure

Subjects were seated individually in private testing rooms after providing informed consent. The experimenter left the room after giving instructions and watching the first few practice trials.

Unbeknown to the participants, there were two phases in the experiment. The training phase consisted of 16 blocks of 32 trials. In each training block, each word was presented once. The training phase was followed by a test phase in which the word-go/stop mapping was reversed (e.g., for Participant 1, ‘bamboo’ always occurred on stop trials in the training phase, but it occurred on go trials in the test phase). The test phase consisted of 6 blocks of 32 trials.

In both phases of both conditions, all trials started with the presentation of the colored word in the center of the screen. Half of the participants were instructed to press the space bar of a QWERTY keyboard with the index finger of the dominant hand as quickly as possible when the ink color was green, but to refrain from responding when the ink color was red. The color-go/stop mapping was reversed for the other participants. The word remained on the screen for 750 ms, regardless of go RT in order to equate study time for go and stop stimuli. A response could be given only while the stimulus was on the screen. The intertrial-interval was 750 ms. At the end of each block, the mean RT on go trials, the number of missed responses on go trials, and the number of incorrect responses on stop trials were displayed and subjects had to pause for 10 seconds, after which they could continue by pressing the space bar.

### Results and Discussion

Table B1 shows that go performance in the test phase was comparable to performance in the last six training blocks. This was confirmed by a repeated measures ANOVA that examined the effect of block number (excluding blocks 1–10), *F*(11, 209) = 1.24, *p* = .26, *gen. η^2^ = .016.* Furthermore, we compared go RT in the last training block with go RT in the first test block using a Bayesian *t* test (Rouder, Speckman, Sun, Morey, & Iverson, 2009), and found substantial support for the null hypothesis, B = .23. Thus, go performance was not influenced by the reversal of the word-go/stop mapping. Again, we attribute the absence of an effect of associative learning on performance to the interplay between attention and automaticity. In this experiment, we did not measure expectancy.

P(respond|signal) was very low in both the training phase and the test phase (Table B1), so we did not analyze it further. [Table-anchor tbl6]
