# Correction to Best et al. (2016)

**DOI:** 10.1037/xhp0000366

**Published:** 2017-02

In the article “Should I Stop or Should I Go? The Role of Associations and Expectancies” by Maisy Best, Natalia S. Lawrence, Gordon D. Logan, Ian P. L. McLaren, and Frederick Verbruggen (*Journal of Experimental Psychology: Human Perception and Performance*, 2016, Vol. 42, No. 1, pp. 115–137. http://dx.doi.org/10.1037/xhp0000116), there is an error in Table 3 of the Results and third paragraph of the **Results** section labeled **Test phase**. In Experiment 4, the study performed an exploratory post-hoc test of the go reaction times in the training phase, contrasting stop-associated and go-associated items. Control items were excluded. Instead of reporting the results of the full analysis (with all three items types included), the authors incorrectly reported the results of this post-hoc analysis in Table 3 and in the main text. The correct analysis is presented below. Note that all other analyses reported in the tables and main text are correct. The R code shared via Open Research Exeter data repository (http://hdl.handle.net/10871/17735) is also correct. The interaction between image type and block is no longer significant when control items are included (*p* = .094; *p* = .037 for the post-hoc test). However, this does not alter the conclusion that encouraging subjects to attend to the items influenced retrieval of stimulus-stop associations: the authors still found a reliable effect of item type in the *p*(respond|stop) measure during training, a reliable effect of item type during the test phase for go reaction times, and a numerical trend in the test phase for the *p*(respond|stop) measure. The numerical trends in go RT during training (see Figure 6) are also consistent with the idea that learning influenced performance in this task. The correct table is presented below.[Table-anchor tbl3]

## Figures and Tables

**Table 3 tbl3:** Overview of Repeated Analyses of Variance Performed to Compare Go and Stop Training Phase Performance

Experiment/factor	*df* = 1	*df* = 2	SS1	SS2	*F*	*p*	η^2^
Experiment 1							
Go reaction time							
Image type	2	56	21980	41878	14.70	<0.001	0.009
Block	11	308	43427	1438376	0.85	0.575	0.017
Image type × Block	22	616	17475	431981	1.13	0.331	0.007
*p*(respond|stop)							
Image type	1	28	0.071	0.323	6.17	0.019	0.005
Block	11	308	0.547	7.616	2.01	0.043	0.040
Image type × Block	11	308	0.154	3.384	1.28	0.238	0.011
Experiment 2							
Go reaction time							
Block	11	319	27502	405062	1.97	0.039	0.037
*p*(respond|stop)							
Block	11	319	0.199	3.846	1.50	0.136	0.037
Experiment 3							
Go reaction time							
Image type	2	60	5589	47836	3.51	0.058	0.006
Block	5	150	64257	437275	4.41	<0.001	0.061
image type × block	10	300	16048	162531	2.96	0.005	0.016
*p*(respond|stop)							
Image type	1	30	0.010	0.200	1.48	0.232	0.002
Block	5	150	0.053	2.688	0.60	0.703	0.009
Image type × Block	5	150	0.055	1.780	0.93	0.461	0.009
Experiment 4							
Go reaction time							
Image type	2	54	6816	87255	2.11	0.154	0.009
Block	5	135	75824	192102	10.66	<0.001	0.093
Image type × Block	10	270	13700	203294	1.82	0.094	0.018
*p*(respond|stop)							
Image type	1	27	0.087	0.317	7.45	0.011	0.015
Block	5	135	0.109	1.857	1.58	0.173	0.019
Image type × Block	5	135	0.180	2.363	2.05	0.077	0.031
*Note*. Image type (Experiments 1, 3, and 4: stop-associated, go-associated, control) and block (Experiments 1 and 2: 1–12; Experiments 3 and 4: 1–6) are the within-subjects factors. We did not analyze *p*(miss) because values were low. SS = sum of squares.							

