# Supplementary material for: Should I Stop or Should I Go? The Role of Associations and Expectancies
Source: J Exp Psychol Hum Percept Perform. 2015 Aug 31;42(1):115–37. doi: 10.1037/xhp0000116 (PMC4685931; doi:10.1037/xhp0000116)

**Supplementary Material**

**Outlier Data**

Subjects were excluded who incorrectly executed a response on ≥ 30% of the stop-signal trials. In Experiment 1, two subjects were excluded; in Experiment 3, one subject was excluded; in Experiment 4, four subjects were excluded. No subjects were excluded from Experiment 2. Exclusion of these subjects did not substantially alter the overall pattern of data. The descriptive statistics with these subjects are presented in Tables S1 and S2.

**Table S1:** Probability of a response on a stop-signal trial [*p*(respond|stop] and RT on go trials as a function of experiment, image type and experimental phase. M = mean; sd = standard deviation.

|  |  | *p*(respond\|stop) | | | | *Go RTs* | | | |
| --- | --- | --- | --- | --- | --- | --- | --- | --- | --- |
|  |  | *Training phase* | | *Test phase* | | *Training phase* | | *Test phase* | |
|  |  | *M* | *sd* | *M* | *sd* | *M* | *sd* | *M* | *sd* |
| Experiment 1 | |  |  |  |  |  |  |  |  |
| stop-associated | | 0.149 | 0.152 | - | - | 410 | 61 | 392 | 43 |
| go-associated | | - | - | 0.175 | 0.164 | 399 | 48 | 392 | 39 |
| control | | 0.167 | 0.169 | 0.188 | 0.185 | 402 | 47 | 388 | 45 |
| Experiment 3 | |  |  |  |  |  |  |  |  |
| stop-associated | | 0.124 | 0.127 | - | - | 428 | 54 | 413 | 46 |
| go-associated | | - | - | 0.182 | 0.153 | 421 | 37 | 406 | 40 |
| control | | 0.134 | 0.156 | 0.125 | 0.119 | 421 | 39 | 410 | 39 |
| Experiment 4 | |  |  |  |  |  |  |  |  |
| stop-associated | | 0.177 | 0.136 | - | - | 450 | 51 | 435 | 50 |
| go-associated | | - | - | 0.263 | 0.183 | 438 | 40 | 418 | 36 |
| control | | 0.214 | 0.174 | 0.215 | 0.209 | 442 | 39 | 420 | 31 |

**Table S2:** Expectancy ratings as a function of experiment and image type. M = mean; sd = standard deviation.

|  |  | *Training phase* | | *Test phase* | | *End of task* | |
| --- | --- | --- | --- | --- | --- | --- | --- |
|  |  | *M* | *sd* | *M* | *sd* | *M* | *sd* |
| Experiment 1 |  |  |  |  |  |  |  |
| stop-associated | | - | - | - | - | 4.88 | 1.47 |
| go-associated | | - | - | - | - | 3.99 | 1.27 |
| control | | - | - | - | - | 4.32 | 1.25 |
| Experiment 3 |  |  |  |  |  |  |  |
| stop-associated | | - | - | - | - | 5.54 | 1.31 |
| go-associated | | - | - | - | - | 4.62 | 0.94 |
| control | | - | - | - | - | 4.79 | 0.93 |
| Experiment 4 |  |  |  |  |  |  |  |
| stop-associated | | 4.52 | 1.15 | 4.77 | 1.15 | - | - |
| go-associated | | 4.06 | 1.13 | 3.91 | 1.08 | - | - |
| control | | 4.23 | 1.03 | 4.02 | 0.91 | - | - |

**RT Percentiles**

To investigate the possibility that the absence of an effect of image type in the test phase of Experiments 1-3 is due to response latencies (responding was faster in the test phase than in the training phase) we plotted RT percentiles for the training and test phases. These RT percentiles revealed that the overall response latency cannot account for the absence of a learning effect in the test phase.

Furthermore, in Experiments 1-3, visual inspection of the percentile plots suggests that the slowing for the stop-associated images emerges in the slow end of the RT distribution. This conclusion is supported by a reliable two-way interaction between image type (stop; go; control) and percentile in the training phase of Experiment 1, *F*(4, 112) = 8.03, *p* = .001, *gen. η2* = .005. However, in Experiment 4, the slowing for the stop-associated images emerges in the fast end of the RT distribution. This conclusion is also supported by a reliable two-way interaction between image type and percentile in the training phase, *F*(4, 108) = 28.05, *p* < .001, *gen. η2* = .034.

In Experiments 1-3, processing the image could slow overall RT; but for stop-associated items, processing the image would also lead to retrieval of the stop associations, and consequently, automatic inhibition of the response. Alternatively, only on slower trials, the stimulus-stop associations could be retrieved in time and affect performance. In Experiment 4, attention to the images prior to signal presentation meant that there was more time for the acquired stimulus-stop associations to be retrieved and thus influence performance.

Figure S1: go RTs (in ms) in the training phase (blocks 1-12; upper panel) and the test phase (blocks 13-14; lower panel) for the three image types (stop-associated; go-associated; control) as a function of percentile in Experiment 1.


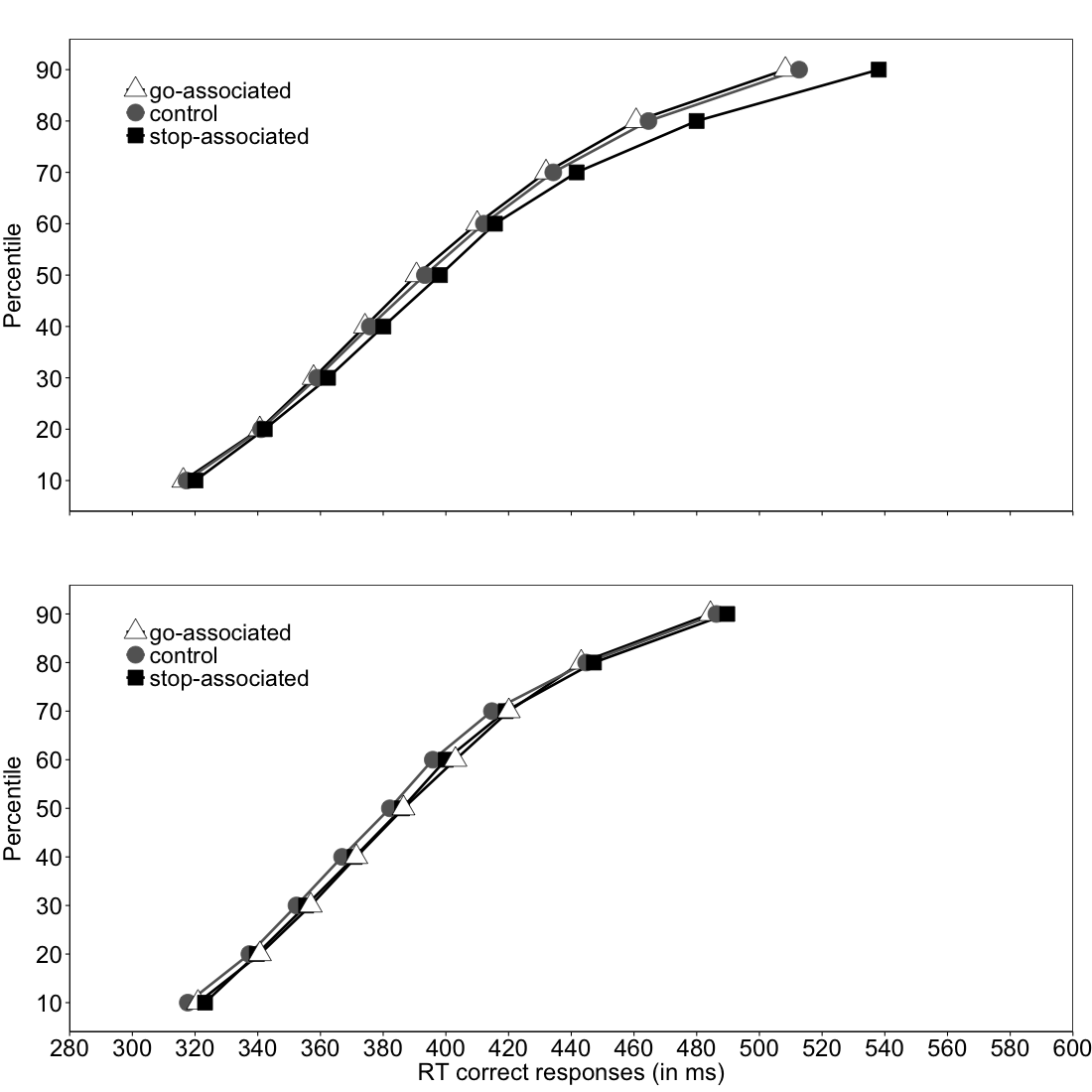


Figure S2: go RTs (in ms) in the test phase (blocks 13-14) for the two image types (stop-associated; go-associated) as a function of percentile in Experiment 2. For obvious reasons, we could not plot RT percentiles for the training phase data.


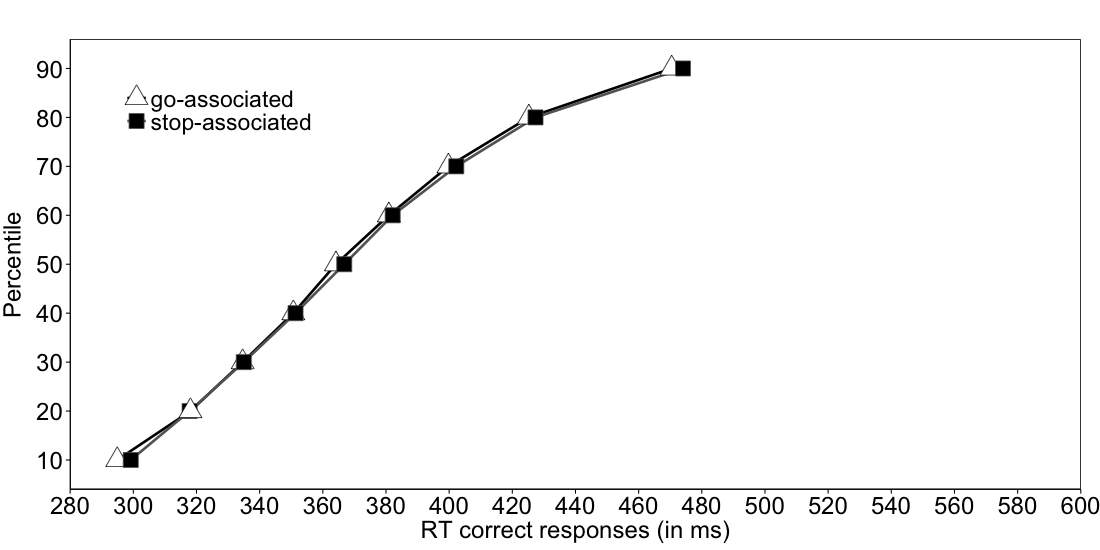
Figure S3: go RTs (in ms) in the training phase (blocks 1-6; upper panel) and the test phase (block 7; lower panel) for the three image types (stop-associated; go-associated; control) as a function of percentile in Experiment 3.


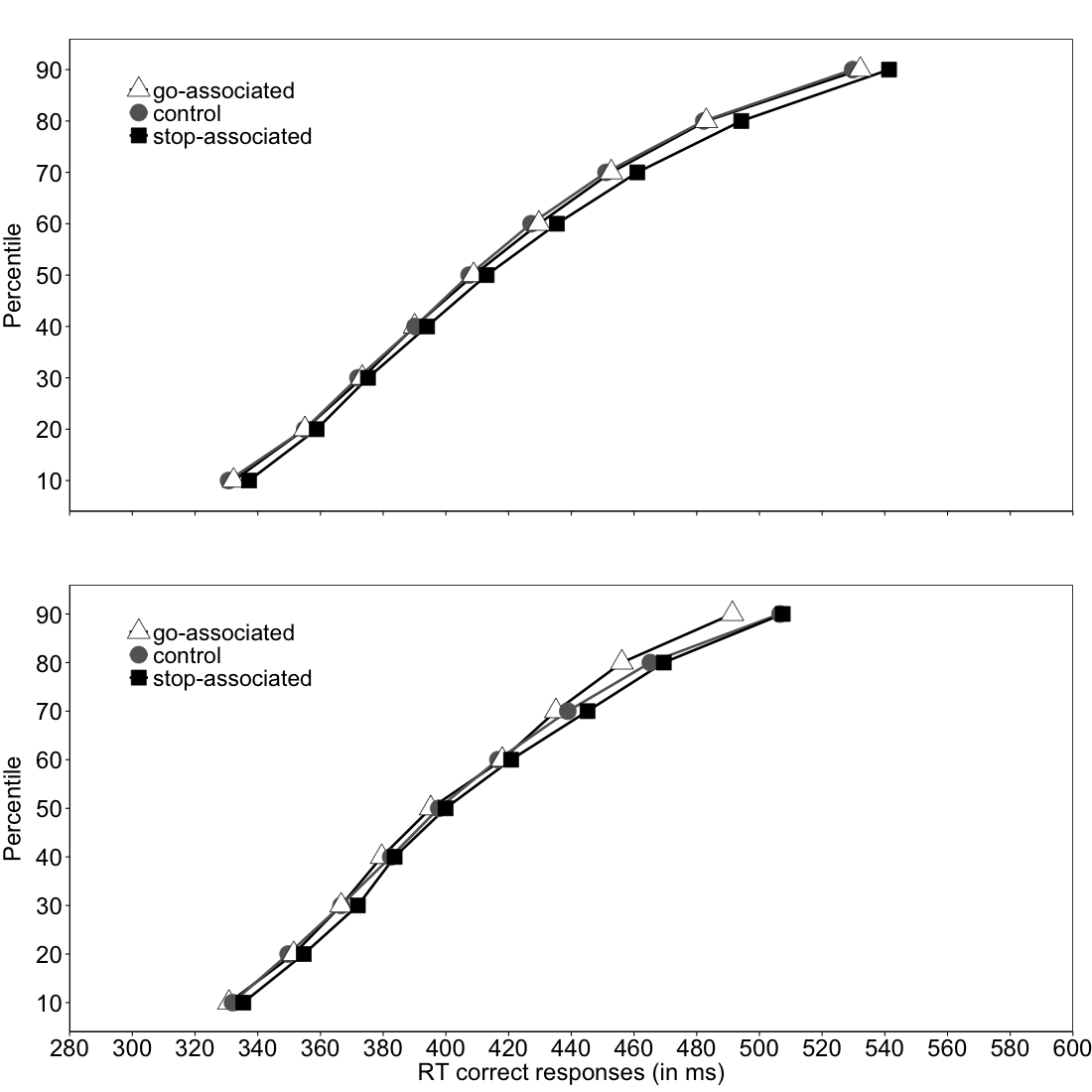
Figure S4: go RTs (in ms) in the training phase (blocks 1-6; upper panel) and the test phase (block 7; lower panel) for the three image types (stop-associated; go-associated; control) as a function of percentile in Experiment 4.


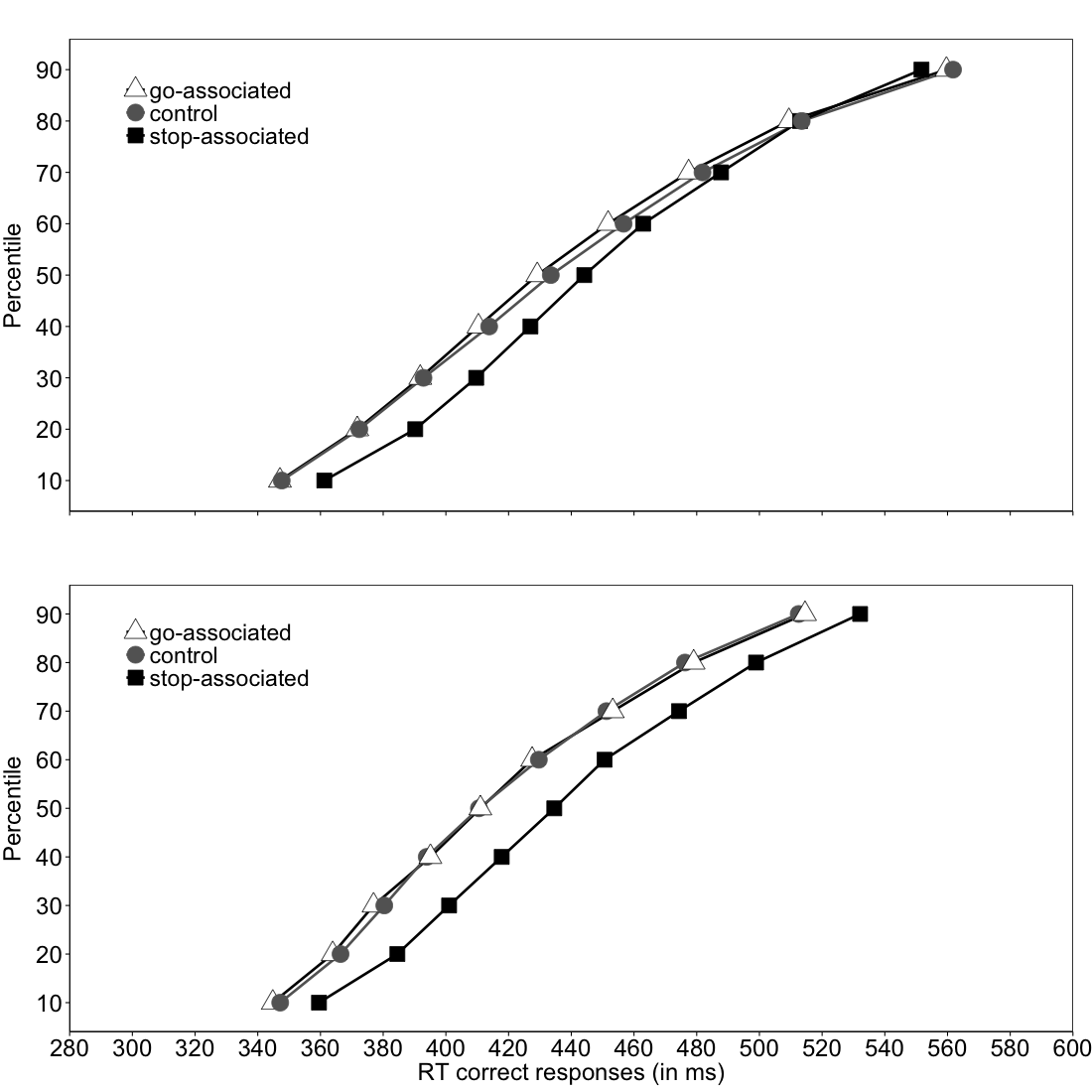
**Expectancy/RT correlation plots**

Figure S5: Expectancy/RT correlations in the training phase (blocks 1-12; upper panels) and the test phase (blocks 13-14; lower panels) in Experiment 1. Note, ‘stop-associated minus control image’ expectancy difference reliably correlated with the corresponding RT difference in the test phase, *r*(26) = 0.437, *p* = 0.019. All other correlations were not reliable (*r*’s ≤ 0.272, *p’s* ≥ 0.161).


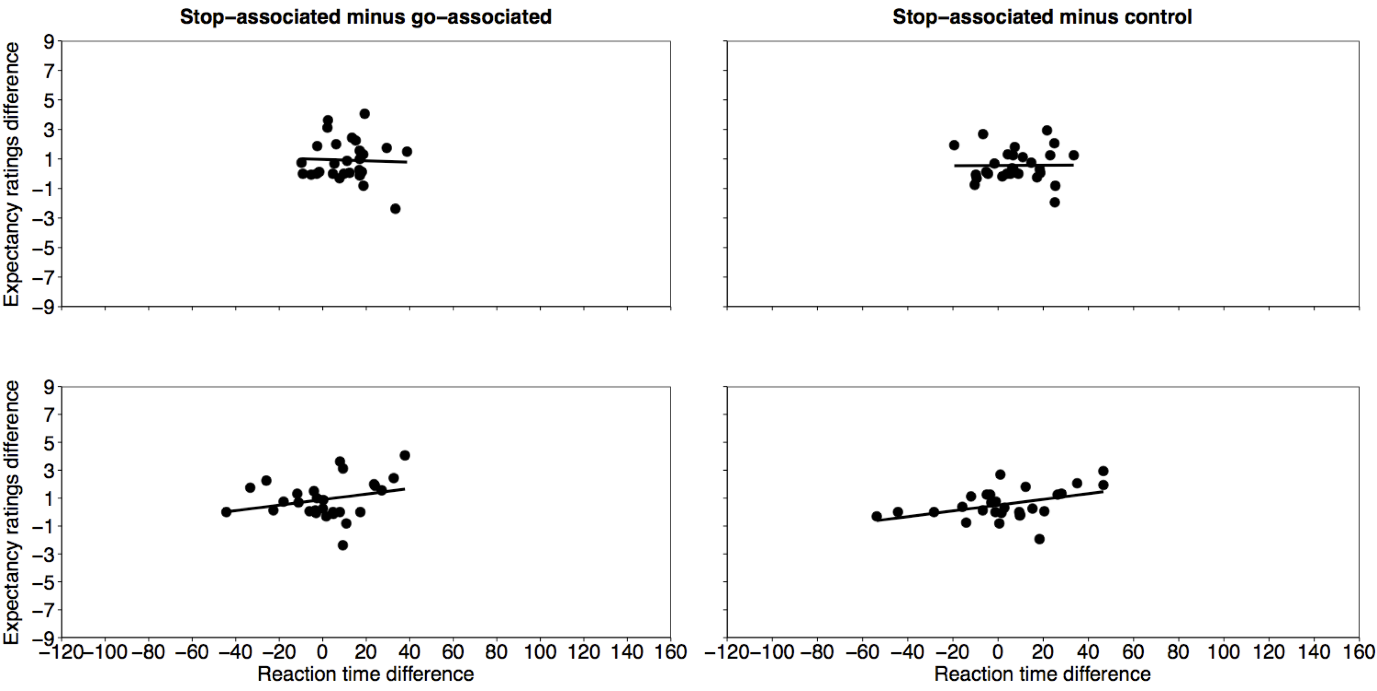
Figure S6: Expectancy/RT correlations in the test phase (blocks 13-14; lower panel) in Experiment 2. Due to the stimulus-stop contingencies used, we could not run these correlations on the training phase data. Note, the stop-associated minus go-associated correlation was not reliable (*r*(28) = 0.262, *p* = 0.162).

**
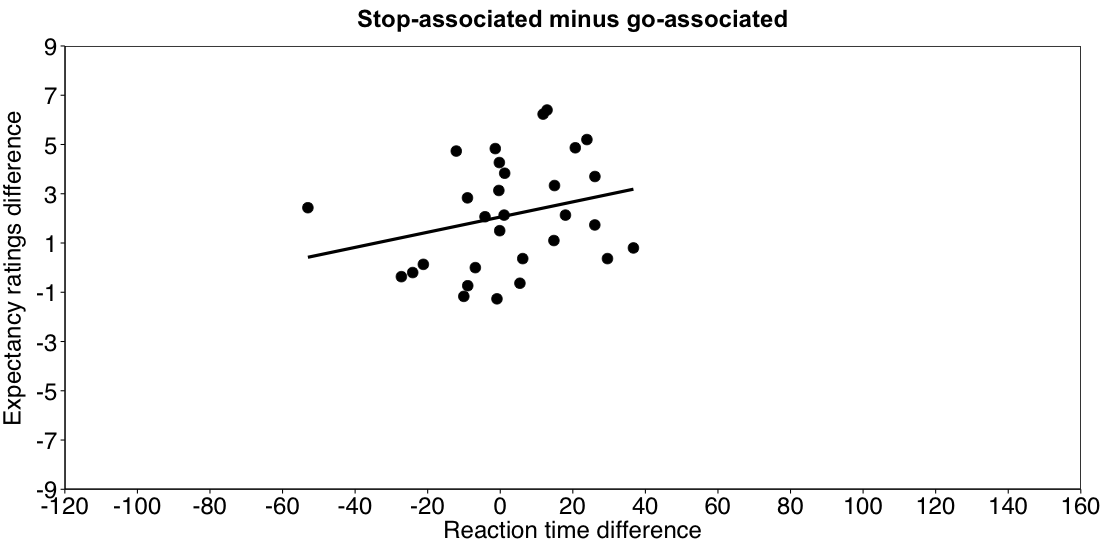
**Figure S7: Expectancy/RT correlations in the training phase (blocks 1-6; upper panels) and the test phase (block 7; lower panels) in Experiment 3. Note, all correlations were not reliable (*r*’s ≤ 0.136, *p’s* ≥ 0.464).


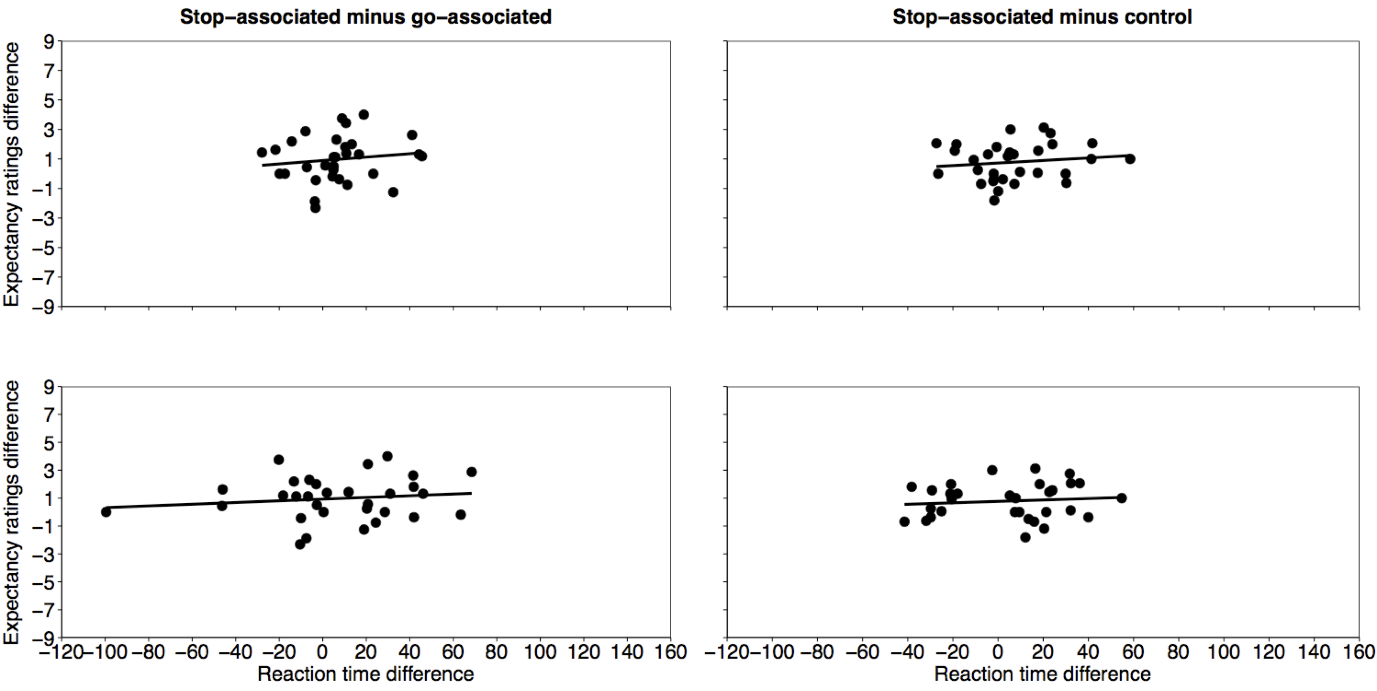
Figure S8: Expectancy/RT correlations in the training phase (blocks 1-6; upper panels) and the test phase (block 7; lower panels) in Experiment 4. Note, all correlations were reliable (*r*’s ≥ 0.498, *p’s* ≤ 0.006).


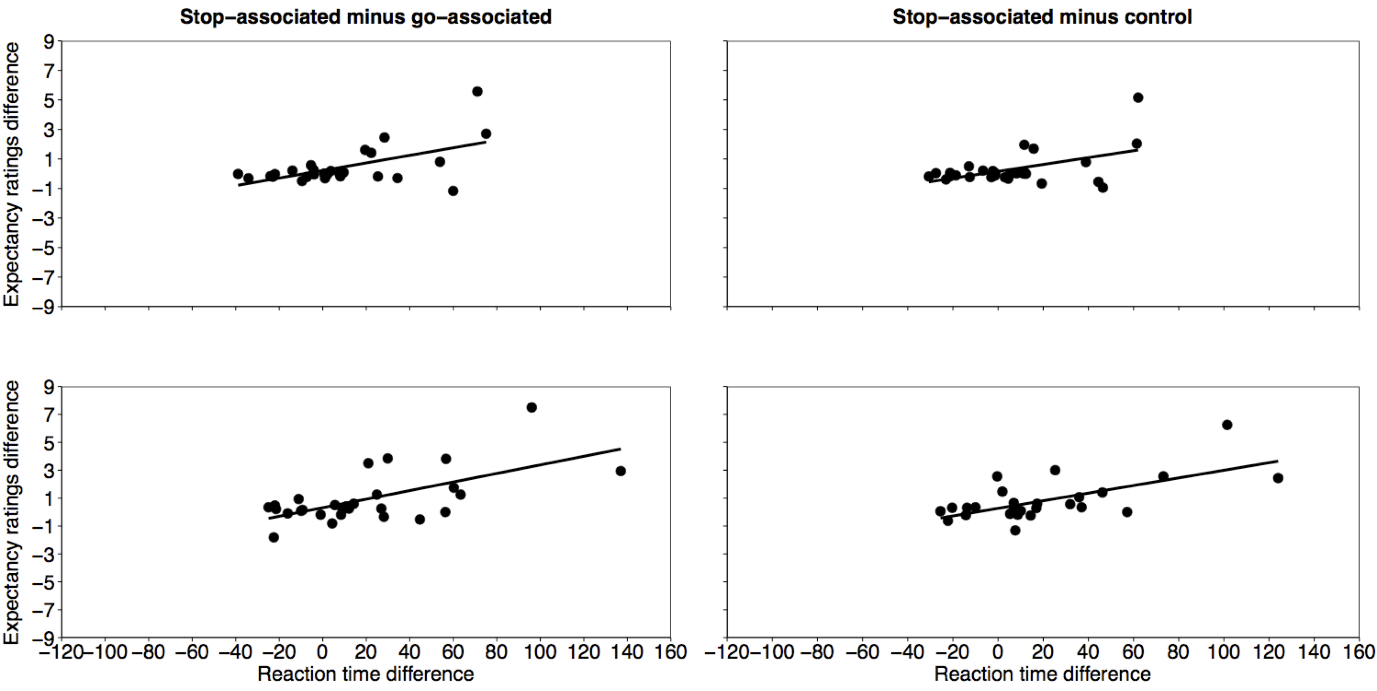

Supplement: Supplementary file 1 [file XHP-2015-0355-Supplementary-Material.docx]
